# Physiotherapists are underutilised in health-sector responses to gender-based violence: A cross-sectional survey of preparedness across Spanish-speaking countries

**DOI:** 10.1371/journal.pgph.0007075

**Published:** 2026-09-02

**Authors:** María Eugenia de León Pérez, Moisés Jonathan Magos Chong, Maira Alejandra Gamboa Gutiérrez, Jimena Figueroa Valero, Raquel García Tarrazo, Denis Montoya-Valverde, Paulina Nicole Cedas Ravena, Carmen María Gantenbein Leiva, Manuel Alexander Gonzales Odar, Jorgelina Dellacasa, Claudia Belen Aguilar Ruiz Velasco, Ana Catalina Mejía de Guardado, Grace McKeon, Davy Vancampfort, Megan Teychenne, Uzma Choudhry, Thea Baker, Simon Rosenbaum

**Affiliations:** 1 Faculty of Health Sciences, Universidad Anáhuac México, Mexico City, Mexico; 2 Independent Physiotherapist, Cali, Colombia; 3 Independent Physiotherapist, Avilés, Spain; 4 Scientific Society of Physiotherapy in Mental Health, College of Therapists of Costa Rica, San José, Costa Rica; 5 Independent Physiotherapist, Coyhaique, Chile; 6 Mental Health Physiotherapy Association of Guatemala (ASOFISME), Guatemala City, Guatemala; 7 Physical Medicine Service, Hospital de Alta Complejidad Virgen de la Puerta, Trujillo, Peru; 8 Kinesiology and Physiotherapy Program, Universidad del Gran Rosario (UGR), Rosario, Argentina; 9 Faculty of Medicine, University of El Salvador, San Salvador, El Salvador; 10 Nutrition, Exercise & Social Equity (NExuS) Research Group, Discipline of Psychiatry and Mental Health, School of Clinical Medicine, University of New South Wales, Sydney, Australia; 11 KU Leuven Department of Rehabilitation Sciences, KU Leuven, Leuven, Belgium; 12 Institute for Physical Activity and Nutrition (IPAN), School of Exercise and Nutrition Sciences, Deakin University, Geelong, Australia; PLOS: Public Library of Science, UNITED STATES OF AMERICA

## Abstract

Gender-based violence (GBV) is a major global public health and human rights issue with wide-ranging physical and mental health consequences. Physiotherapists frequently work with populations and conditions plausibly linked to violence exposure, yet little is known about their preparedness to identify and respond to GBV in Spanish-speaking contexts. We conducted a cross-sectional online convenience survey of Spanish-speaking physiotherapists from Latin America and Spain using the Spanish-language Physician Readiness to Manage Intimate Partner Violence Survey (PREMIS), originally developed and validated for intimate partner violence (IPV) and administered here with broader GBV framing (violencia basada en género) while retaining some IPV-specific items. The survey assessed self-reported preparation, self-perceived knowledge, objective knowledge, opinions, and practice issues related to GBV. Descriptive analyses were conducted across PREMIS subscales and item-level responses. A total of 719 physiotherapists from 15 countries were included in the analytic sample. Self-reported preparedness to identify and respond to GBV was low (mean preparation score 2.5/7), as was self-perceived knowledge (2.4/7). Nearly three-quarters of respondents reported not identifying any GBV cases in the previous six months, and engagement in response practices, such as risk assessment, safety planning, documentation, and referral was uncommon. Nearly half (45%) reported no formal GBV training. Respondents also reported limited awareness of institutional protocols, referral resources, and organisational supports for GBV response. Attitudes towards victim-survivors were generally supportive, with low endorsement of victim-blaming beliefs. Physiotherapists in this sample reported low preparedness, limited training, and substantial system-level barriers to responding to GBV in clinical practice. These findings suggest an underused opportunity to strengthen health-sector responses to GBV through physiotherapy education, professional training, and organisational infrastructure that supports safe identification, referral and trauma-informed care. Future research should evaluate strategies for embedding trauma-informed responses to GBV within routine physiotherapy practice.

## Introduction

Gender-based violence (GBV) is a global public health and human-rights crisis. While GBV can affect anyone, it disproportionately affects women and girls [1]. GBV is an umbrella term that encompasses multiple forms of violence, including intimate partner violence (IPV), sexual violence, coercive and controlling behaviours, and violence directed at individuals because of their gender identity or sexual orientation [2]. The most common form of violence against women globally is IPV and the World Health Organization (WHO) estimates that nearly one in three women worldwide has experienced physical and/or sexual violence in her lifetime, most commonly perpetrated by an intimate partner [1,3].

GBV also affects men and people of diverse sexual orientations and gender identities, including LGBTQ+ populations, often with substantial physical and psychological consequences that may be exacerbated by stigma related to identifying as a victim-survivor, minority stress, and barriers to help-seeking [4–6]. These populations remain underrepresented in research and underserved within health systems, perpetuating inequities in recognition, support, and access to care. For health professionals, this underscores the importance of preparedness to identify and respond to different forms of GBV, rather than focusing narrowly on one population or one form of violence.

The health consequences of GBV are wide-ranging across all genders, spanning injury, chronic pain, disability, adverse reproductive and sexual health outcomes, and elevated risks of depression, post-traumatic stress, and suicidality [7]. Longitudinal and cohort evidence also links exposure to IPV and other forms of GBV with cardiometabolic morbidity and all-cause mortality in women [8]. Health systems therefore occupy a critical position in recognition, first-line response, and referral; yet implementation of comprehensive health-sector responses remains uneven and slow, particularly in low- and middle-income contexts [9].

Latin America has among the highest reported rates of GBV and femicide in the world [10,11]. In 2024 alone, at least 3,828 women were killed in gender-related homicides across Latin America and the Caribbean representing approximately 11 per day [12]. Similarly, Spain has recognised GBV as a major public health and social issue, supported by national surveillance systems that document ongoing cases of intimate partner violence and femicide [13]. Regional frameworks, such as the *Inter-American Convention on the Prevention, Punishment, and Eradication of Violence against Women (Belém do Pará Convention)*, explicitly recognize GBV as both a health and human rights priority [14]. Despite this, health systems across the region face substantial resource constraints, and the integration of GBV-related training into professional education and workforce development remains limited [9,15,16]. Notably, most research examining health professionals’ readiness to address GBV has been conducted in high-income, English-speaking contexts, with little evidence from Spanish-speaking countries.

The WHO emphasizes that, for many survivors, health care settings may represent the first or only opportunity to disclose experiences of violence [2]. This underscores the critical role of all health professionals, including allied health practitioners such as physiotherapists, who often engage with patients more frequently and over longer periods than physicians [17]. Physiotherapists commonly manage conditions closely linked to GBV, such as chronic pain, musculoskeletal injuries, and functional impairment, and the therapeutic relationships developed through repeated encounters place them in a unique position to identify, support, and refer survivors [18–20]. However, available evidence which largely comes from high-income, English speaking settings, suggests that physiotherapists and other allied health professionals frequently report low levels of preparedness, limited confidence and uncertainty regarding referral pathways when responding to GBV [21]. Recent commentary has therefore called for the profession to move “beyond bones, joints and muscles” to engage with the social and relational determinants of health, including violence exposure [19,22,23].

While these findings provide important insight, their applicability to Spanish-speaking physiotherapists in Latin America and Spain remains uncertain. The region is characterised by high reported prevalence of GBV, distinct sociocultural norms shaping professional roles, and health professional education systems in which physiotherapy training has traditionally prioritised physical rehabilitation over psychosocial or trauma-informed care. Evidence from Latin America indicates that health professional curricula, including physiotherapy, often lack structured content on violence identification, survivor-centred responses, and referral pathways, with barriers including limited faculty expertise, institutional resistance, and overcrowded curricula [24]. These contextual factors suggest that findings from Australian and other high-income settings may not readily translate, highlighting the need for locally grounded evidence to inform education, practice, and policy responses.

To date, no studies have systematically assessed Spanish-speaking physiotherapists’ knowledge, attitudes, training needs, and clinical practices in relation to GBV, nor examined the prevalence of lived experience within this professional group. At the same time, the main available readiness instruments, including PREMIS were originally developed in relation to IPV. These instruments remain relevant to this question because IPV is one important form of GBV likely to be encountered in health care settings, although they may not fully capture readiness in relation to the broader spectrum of GBV. Understanding physiotherapists’ readiness within Latin America and Spain is therefore a critical first step toward developing contextually relevant education, clinical protocols, and health-system strategies that strengthen the role of physiotherapy within comprehensive responses to GBV. Accordingly, this study aimed to describe Spanish-speaking physiotherapists’ training, self-reported preparedness, self-perceived knowledge, objective knowledge, opinions, and practice issues relating to GBV in Latin America and Spain.

## Methods

### Ethics statement

Ethical approval for this study was obtained from the Research Committee of the Faculty of Health Sciences, Universidad Anáhuac México (approval reference 202269). All participants were aged 18 years or older and provided written informed consent electronically before commencing the online survey. Participation was voluntary and respondents could stop the survey at any time.

### Study design

This study used a cross-sectional online survey and recruited a convenience sample of Spanish-speaking physiotherapists. Recruitment began November 15, 2024 and ended August 15, 2025. Reporting has been done according to the CHERRIES checklist for reporting on results of internet e-surveys [25].

### Participants and recruitment

Eligible participants were Spanish-speaking physiotherapists aged 18 years or older. Recruitment used convenience sampling and the survey was disseminated through professional physiotherapy networks, academic institutions, and social media platforms widely used in Latin America and Spain, including Facebook, Instagram, LinkedIn, and WhatsApp groups. The mode of dissemination varied across countries, with some recruitment supported by formal organisational endorsement and other recruitment relying primarily on personal and professional networks. Recruitment remained open from November 15, 2024 to August 15, 2025 to allow sufficient time to achieve an adequate sample size and to leverage dissemination opportunities across participating countries. Data were collected and managed using the REDCap platform. The survey link directed participants to a 20–25-minute questionnaire. At the start of the survey, participants were provided with a Participant Information Statement and Consent Form and were required to provide electronic consent before proceeding. Responses were screened for consent status and completeness of demographic and outcome data required for inclusion in the descriptive analyses. Records from participants who did not provide consent were excluded. A separate formal duplicate-detection procedure was not undertaken. Usability and technical functionality were pretested with 11 physiotherapists prior to survey launch, and no substantive changes were required.

### Demographics

Demographic variables collected included age, gender identity (male, female, non-binary or other), country of residence/practice, highest level of education, years in physiotherapy practice, main area of employment (e.g., hospital, private practice, academic), and average weekly hours of paid work. Participants were also asked about prior exposure to GBV training (yes/no), estimated hours of training received, and the context in which training was undertaken (e.g., university curriculum, workplace, professional development, online modules). In addition, participants were asked whether they personally identified as a victim-survivor of GBV. This item was optional and could be skipped without consequence. To minimise potential distress, the question was presented with clear guidance about voluntary disclosure and was accompanied by links to national and international GBV support services available in Spanish. This variable was collected as an optional descriptive characteristic of the sample and was not used as a primary explanatory variable in the analysis.

### Outcome measures

#### Physician Readiness to Manage Intimate Partner Violence (PREMIS) Survey.

The primary outcome was physiotherapists’ readiness to identify and respond to GBV, assessed using the Spanish-language version of the *Physician Readiness to Manage Intimate Partner Violence Survey* (PREMIS) [26,27]. The original instrument and the Spanish adaptation were developed and validated in relation to IPV. In the present study, PREMIS was administered with broader GBV framing to reflect the range of gendered violence that physiotherapists may encounter in clinical practice, while retaining the original IPV-derived item structure, consistent with previous research with allied health professionals [21]. Participants were informed, in Spanish, that the term GBV would be used throughout the survey and was defined according to the United Nations (2023) as acts of harm directed against individuals or groups based on gender, including sexual, physical, psychological, and economic violence, occurring in both public and private settings. No modifications were made to the PREMIS items, response options, subscale structure, or scoring procedures; the adaptation was limited to the introductory framing and terminology. Replacing IPV terminology with broader GBV framing in the introductory instructions may also have influenced how some respondents interpreted individual items, particularly those that were originally developed in relation to partner violence. This approach improved alignment with the study aim, but the findings should be interpreted cautiously, as PREMIS remains an IPV-derived instrument and some items may align more closely with IPV than with the full spectrum of GBV [21,28]. The survey was delivered over five pages, starting with demographics followed by a screen for each of the four PREMIS sub-scales: preparation, knowledge, opinions and practice Issues. Scoring of PREMIS followed the procedures outlined in the original instrument [26]. The PREMIS includes conceptually distinct domains: perceived preparation, perceived knowledge, objective knowledge, opinions, and practice issues/behaviours. In this study, these were analysed separately to distinguish self-reported preparedness and knowledge from objective knowledge, attitudinal responses, and reported clinical practices. A summary of the PREMIS components, item groupings, and scoring approach is provided in Table 1. Summary scores were created for the relevant PREMIS components, with self-reported preparedness and perceived knowledge, and objective knowledge examined as distinct domains alongside opinions and practice issues. Higher scores indicated greater perceived or actual readiness to identify and respond to GBV. In the present sample, internal consistency was high for the perceived preparation items (Cronbach’s α = 0.967) and perceived knowledge items (Cronbach’s α = 0.980). Reliability estimates were calculated for the Likert-type multi-item scales; the objective knowledge and practice issues sections comprised scored knowledge items and heterogeneous behavioural indicators, for which internal consistency estimates were not considered informative in the same way.

**Table 1:** Summary of PREMIS components, item groupings and scoring approach.

PREMIS component	Number of items	Construct assessed	Item type	Scoring/ reporting approach
Perceived preparation	12	Self-reported preparedness to identify and respond to GBV	7-point Likert scale	Reported as mean (SD); higher scores indicate greater self-reported preparedness
Perceived knowledge	15	Self-reported knowledge relevant to responding to GBV	7-point Likert scale	Reported as mean (SD); higher scores indicate greater self-perceived knowledge
Objective knowledge	22	Factual knowledge relating to GBV/IPV-related content	Multiple choice, true/false, and stage-of-change items	Reported primarily using item-level % correct; several items retained IPV-specific framing, so findings are interpreted cautiously.
Opinions	38 (includes added LGBTQ+ item)	Attitudes, beliefs, and self-efficacy regarding GBV response	7-point Likert scale	Reported as domain means (SD) and selected item-level response distributions; negatively worded items reverse-coded where appropriate
Practice issues	27	Self-reported practice behaviours and workplace practices related to GBV	Counts, categorical items, and frequency responses	Reported as n (%) for individual items; higher engagement indicates greater reported clinical response and organisational support

#### Preparation sub-scale.

The Preparation section comprised two related yet distinct self-report components: perceived preparation (e.g., ability to ask about GBV, make referrals, complete documentation) and perceived knowledge (e.g., legal obligations, signs and symptoms, referral resources). Responses were recorded on 7-point Likert scales (strongly disagree to strongly agree), with higher scores indicating greater self-reported preparedness or knowledge.

#### Knowledge sub-scale.

The Knowledge section assessed objective knowledge about GBV/IPV-related content using multiple-choice, true/false, and stage-of-change items covering risk factors, warning signs, reasons survivors may remain in violent relationships, asking about violence, documentation, and legal/reporting issues. Responses were scored as correct/incorrect, with “don’t know” counted as incorrect. Given the heterogeneity of the content covered and the IPV-specific framing of several items, objective knowledge is reported primarily through item-level descriptive analyses.

#### Opinion sub-scale.

The Opinions subscale comprised of 37 items plus one adapted item, assessing attitudes, beliefs, and self-efficacy related to identifying and responding to GBV. Items covered domains such as perceived preparedness, workplace environment, self-efficacy, understanding of victims’ experiences, and views on scope of practice. Responses were recorded on a 7-point Likert scale ranging from 1 (“strongly disagree”) to 7 (“strongly agree”). Negatively worded items were reverse-coded so that higher scores reflected more supportive and positive opinions towards recognising and addressing GBV. The Opinions domains captured staff preparation (perceived readiness and role confidence), legal requirements (knowledge of reporting obligations), workplace issues (perceived organisational support and clinical environment), self-efficacy (confidence in identifying and responding to GBV), alcohol/drugs beliefs, victim understanding, victim autonomy, and staff constraints (perceived time and workload barriers).

In line with previous cultural adaptations of the PREMIS instrument, one item within the Opinions subscale was contextually expanded. Specifically, Item 10 (“I do not have sufficient skills to talk about abuse with a victim of IPV who is…”) retained the original response options referring to women, men, and individuals from different cultural/ethnic backgrounds, and an additional response category was incorporated to assess perceived preparedness when working with individuals from the LGBTQ+ community. This modification was introduced to better reflect the diversity of populations encountered in contemporary clinical practice and to explore potential differences in perceived self-efficacy across victim populations. This additional response option was reported descriptively and was not included in PREMIS domain scoring. Accordingly, comparability with prior PREMIS-based studies was retained for scored domain analyses, although the LGBTQ+ item itself should be interpreted as an exploratory descriptive addition.

Subscale scores were calculated by averaging across items within each domain, with higher scores indicating more favourable attitudes. For descriptive reporting of item-level responses, Item-level percentages reflect the proportion of respondents selecting response options 6 or 7 (agree/strongly agree), or 1 or 2 (disagree/strongly disagree) where appropriate.

#### Practice Issues sub-scale.

The Practice Issues subscale assessed self-reported practice behaviours and workplace practices related to GBV. Items covered the identification of new cases in the past six months, actions taken when GBV was identified (e.g., providing information, safety planning, referral), and follow-up with previously identified cases. Additional items captured the availability of institutional resources and protocols (e.g., referral pathways, documentation tools, photography of injuries, educational materials) and perceptions of whether respondents had adequate knowledge of community referral options. Responses were collected as counts, categorical options, or frequency scales (e.g., never to always). Higher scores indicated greater reported engagement with GBV-related clinical practices.

### Data analysis

Data were analysed using IBM SPSS Statistics version 30.0. Participant demographic characteristics are reported using descriptive statistics, including counts (n) and percentages (%). PREMIS responses were analysed across the four subscales: preparation, knowledge, opinions, and practice issues. The PREMIS domains were examined as distinct constructs, including perceived preparation, perceived knowledge, objective knowledge, opinions, and practice behaviours. Preparation, knowledge, and opinions subscales were treated as continuous variables and are reported as means with standard deviations (SD). Practice issues items were primarily categorical and are reported as counts and percentages. Analyses were conducted on all available data, with missing responses coded as missing and excluded on an item-by-item basis (pairwise deletion), resulting in varying denominators across subscales and items. Pairwise deletion was used to retain as much available information as possible for each descriptive analysis, rather than excluding participants with partial missing data from all analyses. The extent of missingness varied across measures and is reflected in the reported denominators for each subscale and item-level analysis. Consistent with the descriptive aim of the study, analyses focused on summarising levels of preparedness, knowledge, attitudes, and practice related to gender-based violence among physiotherapists across participating countries. Although the overall sample was large, the use of convenience sampling, highly variable subgroup sizes, and differing denominators across items and subscales meant that inferential subgroup comparisons were not considered appropriate. No inferential statistical testing was undertaken to compare subgroups, and results are presented descriptively to characterise patterns within the sample.

## Results

### Participant characteristics

Of the 879 records imported from the survey platform, 860 participants provided consent to take part. Records from participants who did not provide consent were excluded. The final analytic sample comprised 719 physiotherapists with complete demographic data and at least partial outcome data for inclusion in the descriptive analyses. Missing responses within the analytic sample were then retained and handled on an item-by-item basis, resulting in varying denominators across subscales and item-level analyses. Missingness was highest for some PREMIS subscales and item-level responses, resulting in varying denominators across analyses (e.g., n = 600 for perceived preparation, n = 597 for perceived knowledge, n = 533 for objective knowledge, n = 487 for opinions, and n = 454 for practice issues). Thus, the difference between imported records, consenting participants, and the analytic sample reflects exclusion of non-consenting records and records with incomplete demographic data or insufficient outcome data, whereas variation across PREMIS denominators reflects item-level missing responses within the analytic sample. Participants were predominantly female (73%, 26% male and 1% other), with a mean age of 35.3 years (SD 8.9, range 18–79). Given the very small numbers in the non-binary and other gender categories, these responses were combined for descriptive reporting and interpreted cautiously. Most held an undergraduate (63%) or master’s degree (26%). Participants represented multiple countries, with the largest groups from Mexico (19%), Spain (19%), Peru (12%), and Costa Rica (12%). While respondents were drawn from across Latin America and Spain, the relative proportion of respondents was higher in smaller countries (e.g., Guatemala, Costa Rica) and lower in larger countries (e.g., Spain, Argentina, Chile). Therefore, the sample should not be considered nationally representative. The majority were practicing physiotherapists (87%), most commonly working in private practice (44%), hospitals (15%), or community/public health centres (14%) with the remaining 13% comprising physiotherapy educators, researchers or staff in managerial roles. Average weekly employment was most often 30–40 hours (37%). Almost half (43%) had more than 10 years of professional experience.

### Lived experience of Gender-Based Violence

When asked whether they identified as a victim-survivor of any type of GBV, more than one-third of respondents (n = 257, 36%) reported lived experience of GBV, while 34 (5%) preferred not to respond. This variable was included to describe the sample and was not analysed as a primary predictor of preparedness outcomes. These findings indicate that lived experience of GBV was not uncommon within the surveyed physiotherapy workforce and provide important context for interpreting the sample. The proportion reporting lived experience varied by gender: 19% (n = 29) of male physiotherapists, 41% (n = 180) of female physiotherapists, and 71% (n = 5) of those identifying as non-binary or other gender. Given the very small numbers in the non-binary and other gender categories, these patterns should be interpreted cautiously.

### Gender-Based Violence training

Among the 719 respondents, nearly half of (45%) reported having received no formal GBV training, defined here as structured education such as university-based teaching, basic courses, postgraduate training, or advanced/specialised training. Broader GBV-related educational exposure was also reported and included activities such as watching a video, reading a protocol or guideline, or attending a conference or awareness-raising session. The most commonly reported formats were watching a video (24%) or reading a protocol or guideline (19%), followed by attending a conference or awareness-raising session (18%). Less commonly reported formats included theoretical training during a university degree (11%), practical training during a university degree (4%), participation in a basic course of up to 20 hours (10%), or advanced/specialised training (2%). In terms of dose, when asked to estimate the total number of hours of training received (n = 606), 15% reported less than 1 hour of training, 17% reported 1–3 hours, and 9% reported more than 10 hours. Training was most commonly received in the workplace (19%) or at university (18%), with a further 15% reporting informal or self-directed learning (e.g., social media or online platforms).

A summary of participant characteristics, lived experience of gender-based violence, and gender-based violence training is provided in Table 2.

### Self-reported preparedness sub-scale

Overall self-reported preparedness scores were low on the 7-point scale, with a mean score of 2.5 (SD = 1.29, n = 600). Mean preparedness scores differed slightly by gender, with male physiotherapists reporting marginally higher scores (M = 2.6, SD = 1.35, n = 153) than female physiotherapists (M = 2.4, SD = 1.26, n = 440). Participants identifying as non-binary or another gender reported higher mean scores (M = 3.3, SD = 1.93), although this subgroup was too small for further analysis. Examination of item-level response distributions demonstrated marked variation in self-reported preparedness across GBV-related clinical tasks (see Table A in S1 Appendix). For enquiry and initial response tasks only 9–10% of respondents rated themselves as “well prepared” or “quite well prepared” (response categories 6 or 7 in the 7-point scale) to ask about GBV, respond to disclosures, or identify indicators of violence. Preparedness was lower for risk assessment and systems-oriented tasks, with 5–7% reporting being well or quite well prepared to conduct lethality assessments, assess risk to children, develop safety plans, document GBV, or make appropriate referrals. Self-reported preparedness was lowest for mandatory reporting tasks where 4% of respondents endorsed the highest preparedness categories. Across all preparation items, between 90% and 96% of respondents rated themselves below the “well prepared” threshold (i.e., below response category 6).

### Perceived and objective knowledge

Self-perceived knowledge regarding GBV was low overall (Table B in S1 Appendix). The mean perceived knowledge score was 2.4 (SD = 1.27, n = 597) on the 7-point PREMIS scale. Mean scores differed modestly by gender, with male physiotherapists reporting slightly higher perceived knowledge (M = 2.6, SD = 1.40, n = 153) than female physiotherapists (M = 2.3, SD = 1.21, n = 437). Objective knowledge was also limited and showed substantial variability across item-level domains (Table C in S1 Appendix). Several items within this section retained an IPV-specific framing, and the findings should therefore be interpreted cautiously as an IPV-derived indicator of GBV-related knowledge rather than a comprehensive measure of knowledge across all forms of GBV. Less than half of respondents correctly identified gender as the most important risk factor for experiencing GBV (46%). Regarding the choice of screening questions, 24% correctly identified “Has your partner ever hurt or threatened you,” and 31% correctly identified “Has your partner ever hit or hurt you”. Knowledge regarding perpetrator behaviour was comparatively higher, with 59% correctly identifying violence as a means of control. Recognition of potential clinical indicators of GBV exposure was mixed. While anxiety (81%), frequent injuries (79%), and depression (77%) were commonly identified as warning signs, fewer respondents recognised substance abuse (47%) or chronic pain without an apparent cause (56%) as potential indicators. Understanding of survivor behaviour was limited: although individual reasons for remaining in violent relationships, such as financial dependence (89%), fear of retaliation (88%), and concern for children (79%) were frequently identified, only 27% correctly identified all contributing factors. Knowledge relating to survivor-centred care and clinical processes also varied. Most respondents correctly recognised that many survivors present without visible injuries (71%) and that leaving a violent relationship may increase risk (51%). However, fewer than one-third correctly identified all stages in a vignette assessing readiness for change, and only one-third correctly rejected the statement that alcohol consumption is the greatest single predictor of GBV (33%). Correct responses were more common for items relating to documentation practices (64%) and the importance of avoiding coercive advice (61%).

### Opinions sub-scale

Physiotherapists expressed mixed attitudes toward their role and preparedness in responding to GBV (Table D in S1 Appendix). At the domain level, mean scores ranged from 3.4 to 4.6 on the 7-point scale. Scores were lowest for staff preparation (M = 3.4, SD = 1.32) and workplace issues (M = 3.6, SD = 1.14), indicating limited perceived readiness, role clarity, and institutional support. Self-efficacy scores were modest (M = 3.7, SD = 1.22), while scores were higher for victim understanding (M = 4.6, SD = 0.86) and staff constraints (M = 5.1, SD = 1.27) (Table 3).

**Table 2:** Demographic characteristic 719 Spanish-speaking physiotherapists.

Demographic Characteristics		N (%)
Gender		
	Male	186 (26)
	Female	525 (73)
	Non-binary/ Other	8 (1)
Age – Mean (SD)		35.27 (8.9) Range 18–79
Age groups (years)		
	18 – 25	78 (11)
	26 – 34	284 (39)
	35 – 44	253 (35)
	45 – 54	84 (12)
	>=55	20 (3)
		
Highest education level		
	PhD	26 (4)
	Undergraduate	456 (63)
	Masters	183 (26)
	Technician	54 (7)
		
Country		
	Mexico	136 (19)
	Spain	126 (19)
	Costa Rica	87 (12)
	Peru	88 (12)
	Chile	76 (11)
	Guatemala	69 (10)
	Argentina	44 (6)
	Colombia	43 (6)
	El Salvador	20 (3)
	Other (Ecuador, Panama, Uruguay, Venezuela etc)	17 (2)
Do you see clients/patients (practicing)? (n = 5 missing)		
	Yes	626 (87)
	No	88 (13)
What are the populations you work with? (yes, (%))		
	Neonates	28 (4)
	Children	105 (15)
	Adolescents	127 (18)
	Adults	499 (69)
	Older adults	390 (54)
How many consults do you provide per week?		
	<20	235 (33)
	20 – 39	241 (33)
	40 - 59	84 (12)
	>60	60 (8)
	Not reported	99 (14)
Principal place of employment		
	Private practice	313 (44)
	Hospital	104 (15)
	Public health/ community health centres	96 (14)
	Academic institution	67 (9)
	Non-for profit	35 (5)
	Corporate	22 (3)
	Gyms	21 (3)
	Sports organisation	13 (1)
	Other (e.g., insurance, emergency services etc)	48 (6)
		
Average hours of paid weekly employment?		
	<10	54 (8)
	10 – 20	70 (10)
	20 – 30	114 (16)
	30 – 40	269 (37)
	>40	212 (29)
		
Time practicing (years)?		
	<1	61 (8)
	1 – 5	155 (22)
	5 – 10	191 (27)
	>10	312 (43)
		
Do you identify as a victim-survivor of gender based violence?		
	No	428 (59)
	Yes	257 (36)
	Prefer not to respond	34 (5)
		
	Males	29 (19%)
	Females	180 (41%)
	Other	5 (71%)
What type of training have you had on GBV issues? Select all that apply.		
	I have read protocols or guidelines	139 (19)
	I have watched a video	171 (24)
	I have attended a conference or awareness raising session	127 (18)
	I have participated in a basic course (up to 20 hours)	68 (10)
	I received theoretical training during my degree	80 (11)
	I received practical training during my degree	27 (4)
	Training during residency or postgraduate studies	25 (4)
	Advanced-specialized training	12 (2)
	Another in-depth training (more than 20 hours)	20 (3)
	Other	257 (36)
		
Estimated total number of GBV training hours received (n = 113 missing)	None	272 (45)
	<1	90 (15)
	1 – 3	104 (17)
	3 – 5	49 (8)
	5 – 10	39 (6)
	>10	52 (9)
	missing	113
		
Where was training received?	University	127 (18)
	Workplace	139 (19)
	Other (e.g., social media, YouTube)	109 (15)
		

**Table 3:** Opinions sub-scales (N = 487).

Item	Mean	SD.
Staff preparation	3.4	1.32
Legal requirements	3.9	1.86
Workplace issues	3.6	1.14
Self-efficacy	3.7	1.22
Alcohol/ drugs	4.2	0.74
Victim understanding	4.6	0.86
Victim autonomy (not used final version*)	4.3	1.07
Constraints	5.1	1.27

At the item-level, 43% of respondents agreed or strongly agreed that they lacked sufficient preparation to help clients experiencing GBV, while only 11% actively disagreed with this statement (Table D in S1 Appendix). Perceived skills deficits were reported across multiple client groups, with 28–31% of respondents agreeing or strongly agreeing that they lacked sufficient skills to discuss abuse with female, male, culturally diverse, and LGBTQ+ clients (Table D in S1 Appendix). Confidence in initiating conversations about GBV was low, with only 18% of respondents agreeing or strongly agreeing that they felt comfortable talking about GBV with clients. Perceived legal knowledge was low, with only 4–7% agreeing or strongly agreeing that they knew mandatory reporting requirements for GBV, elder abuse, or child abuse. Most respondents disagreed with the overtly victim-blaming statements, and scores relating to victim understanding were comparatively more supportive. Full item-level response distributions for the Opinions sub-scale are presented in Table D in S1 Appendix.

### Practice sub-scale

Engagement with GBV-related clinical practice was limited across four areas: case identification, clinical response following identification, institutional resources, and educational/referral materials (Table E in S1 Appendix). Most physiotherapists reported not identifying any cases of GBV in the past six months (74%), with an additional 6% indicating that they did not work in a clinical setting. Among those who had identified at least one case, reported clinical responses such as referral, safety planning and risk assessment were uncommon. Institutional systems to support GBV-related practice were also inconsistently reported and availability and use of educational and referral materials was limited. Only 7% indicated that their service had a protocol for managing GBV that was routinely used, while a further 11% reported the existence of a protocol that was used to some extent. In contrast, 40% reported that no protocol was available, and 27% were unsure whether such a protocol existed. Consistent with this, most respondents (67%) reported that they were not familiar with their institution’s policies or programs for the detection and management of GBV. Access to practical resources was also limited, with only 17% reporting the availability of a camera to document injuries. Among respondents who indicated that the items were applicable to their practice, engagement in GBV-related clinical actions was generally infrequent (Table E in S1 Appendix). For most documentation and safety-related practices, approximately 45% of respondents reported never performing the action, including documenting a client’s testimony in the medical record (38%), using a body chart to document injuries (43%), photographing injuries (47%), conducting a risk assessment of the victim (40%), assessing risk to the victim’s children (42%), and assisting with safety planning (39%). In each of these domains, fewer than 10% of respondents reported performing the action almost always or always. Referral-related practices were similarly uncommon. Nearly 40% of respondents reported never contacting a service provider for GBV victims (39%), while only 11% reported doing so almost always or always. Provision of basic information about GBV and referral information for other resources showed slightly higher engagement but remained limited, with approximately one-third reporting never providing this information and fewer than 15% reporting that they did so almost always or always. Accordingly, low reported action in this section may reflect a combination of non-identification, limited opportunity to apply these practices, low confidence or competence, and system-level barriers, rather than any single explanation. This is particularly important given the high proportion of “not applicable” responses and the inclusion of respondents who were not currently working in clinical roles or relevant practice settings.

In contrast, supportive interpersonal responses were more commonly endorsed. Offering validating or supportive communication was the most frequently reported action, with 21% of respondents indicating that they performed this almost always or always, although 29% still reported never doing so. Across all items, a substantial proportion of respondents (38–39%) selected “not applicable”. This likely reflects both that many physiotherapists had not encountered situations in which these GBV-related practices could be applied during the reference period (Table F in S1 Appendix) and that some respondents were not currently engaged in clinical roles or in settings where particular practices were relevant. Low reported engagement should therefore be interpreted cautiously and not assumed to reflect lack of action alone. Availability and use of educational and referral resources for GBV were limited (Table G in S1 Appendix). More than half of respondents (58%) reported that no GBV-related educational materials (e.g., leaflets, posters, information) were available in their service, while only 8% reported that such materials were clearly visible and accessible to clients. Provision of educational materials to clients experiencing GBV was uncommon: only 5% reported providing materials almost always, and 9% reported doing so when it was safe for the client. In contrast, 32% reported not providing materials due to a lack of adequate referral resources in the community. Consistent with this, perceived access to referral pathways was low, with only 9% of respondents reporting that they had appropriate referral resources for GBV cases in their community, while 53% reported that they did not and 21% were unsure.

## Discussion

This study found low levels of self-reported preparedness, limited training, infrequent identification of GBV, and substantial system-level barriers to response among physiotherapists across 15 Spanish-speaking countries in Latin America and Spain. Together, these findings suggest a gap between potential contribution of physiotherapy to health-sector responses to GBV and current clinical and organisational realities. This is important given the high prevalence and health burden of GBV globally [29,30].

The low level of engagement in identifying and responding to GBV observed in this study was accompanied by low levels of self-reported preparedness, self-perceived knowledge, and confidence across multiple domains. Participants reported limited preparedness to ask about GBV, respond to disclosures, conduct risk assessments, document violence, and make appropriate referrals, with fewer than one in five endorsing confidence in initiating conversations about violence. Objective and perceived knowledge were also limited, including poor understanding of screening approaches, referral resources, and legal reporting requirements. These patterns were observed alongside limited awareness of institutional protocols, uncertainty regarding referral pathways, and lack of confidence in available resources. Taken together, the findings suggest that low identification and response to GBV in physiotherapy practice may be shaped by educational, organisational and professional-role factors, although these mechanisms were not directly tested in the present study.

These findings are consistent with emerging evidence from allied health professionals in other settings [18,19,21], which similarly highlights low levels of preparedness, confidence, and system support for responding to GBV in clinical practice. Evidence from Australia reported that allied health professionals frequently recognise the relevance of GBV to their work but feel underprepared to ask about violence, uncertain about referral pathways, and constrained by limited training and organisational support [21]. The present study extends this evidence in several important ways. First, it focuses specifically on physiotherapists, a profession characterised by frequent and sustained client contact and management of conditions closely aligned with the health sequelae of GBV. The nature of physiotherapy practice in particular, involving close physical proximity, touch, and activities that may trigger trauma responses, places physiotherapists in a unique position to recognise signs of violence, provide supportive care, and facilitate appropriate referral, highlighting the importance of GBV awareness and training for safe and effective practice [19].

Second, it provides the first large-scale, multi-country data from Spanish-speaking contexts in Latin America and Spain, addressing a notable gap in a literature that has been dominated by high-income, English-speaking settings. Finally, by using the Spanish-language PREMIS instrument originally validated in relation to IPV [27], this study extends existing allied health research into Spanish-speaking contexts. However, given the IPV-derived basis of the instrument and the broader GBV framing used here, the findings are best interpreted as evidence of an important readiness gap in this sample rather than as a definitive measure of allied health readiness across the full spectrum of GBV.

Interpretation of these findings should be considered in light of the measurement framework used. Although the PREMIS instrument was originally developed to assess readiness to manage intimate partner violence, the present study administered the Spanish-language version with broader GBV framing. This decision reflects both the terminology embedded in the Spanish PREMIS and the reality that physiotherapists may encounter multiple forms of gendered violence in practice, including sexual violence, coercive and controlling behaviours, and violence outside intimate partnerships. However, several objective knowledge items within PREMIS retain an IPV-specific framing, particularly those relating to risk factors and screening approaches. As a result, knowledge relevant to non-partner forms of GBV may be underestimated. This highlights an important gap in existing assessment tools and underscores the need for future research to develop and validate measures that more fully capture allied health professionals’ readiness to respond to the broader spectrum of gender-based violence encountered in contemporary clinical settings.

The findings of this study have clear implications for physiotherapy education and professional training. Across all PREMIS domains, respondents reported minimal exposure to GBV-related training, with most reporting either no formal training or only brief, ad hoc exposure through passive formats such as videos or written materials. Training integrated into undergraduate or postgraduate physiotherapy curricula was uncommon, and confidence in core clinical skills, including asking about violence, responding to disclosures, assessing risk, and making referrals, was consistently low. These findings suggest that current educational pathways may not be adequately preparing physiotherapists to engage safely and effectively with GBV in practice.

There is emerging evidence that trauma and violence-informed (TVI) physical activity training can strengthen service provider capability. TVI approaches recognise the pervasive impact of trauma and violence on health, prioritise safety, trust, choice and empowerment, and aim to minimise the risk of re-traumatisation within service delivery [31,32]. In the context of GBV, this framework is particularly relevant, as physiotherapists may work with individuals whose trauma histories are not disclosed but influence engagement, communication, and therapeutic relationships. An evaluation of online training modules for exercise professionals, community service providers and other psychosocial professionals found significant improvements in knowledge, confidence and the application of trauma informed principles [33]. While this demonstrates the feasibility and potential impact of such training, it was not specifically designed for physiotherapists, whose broader clinical scope requires more tailored educational needs [20]. Therefore, embedding structured, skills-based GBV training within physiotherapy education may represent a critical opportunity to address this gap. Such training should extend beyond awareness-raising to include trauma-informed communication, recognition of health presentations associated with violence, clarification of professional roles and boundaries, and practical guidance on referral pathways and interprofessional collaboration. To be effective, such training must be context-responsive, aligning with local legal frameworks, cultural norms, and available health and social service systems, and supported by clear professional guidelines that delineate scope of practice and referral responsibilities across different country contexts [34]. Importantly, given the complexity of GBV and the sensitive, relational skills required to respond appropriately, training should emphasise opportunities for skills practice and reflection, alongside ongoing professional development to maintain competence over time. Taken together, the findings suggest that these educational gaps are not limited to one setting and support the need for curriculum development and continuing professional education that reflects the realities of contemporary physiotherapy practice.

Beyond individual knowledge and training gaps, the findings point to substantial system-level barriers that constrain physiotherapists’ ability to respond to GBV in practice. Most respondents reported limited awareness of institutional policies, protocols, or programs for the detection and management of GBV, alongside uncertainty regarding referral pathways and available community resources. Routine practices that support a coordinated health-sector response such as documentation, risk assessment, safety planning, and referral were uncommon, and few respondents reported working in services with established or actively used GBV protocols. These findings align with broader evidence indicating that health professionals’ responses to GBV are heavily shaped by organisational context, and that training alone is insufficient in the absence of enabling systems [2,9,35]. Time pressures, workload demands, and lack of institutional support were frequently endorsed as constraints, suggesting that organisational context may play an important role in shaping whether clinicians feel able to respond to GBV in practice. Strengthening system-level supports within health services and professional bodies may therefore be critical to translating improved education and training into sustained changes in clinical practice. Future qualitative research would also help to understand how professional identity, organisational culture, and perceived scope of practice shape physiotherapists’ responses to GBV.

## Reflexivity and research partnership

In line with recent calls for greater reflexivity and transparency in global health research partnerships [36,37], we note that this study was shaped by collaboration between physiotherapists and non-physiotherapist researchers working across Latin America, Spain and Australia. The initial study concept emerged from prior work using PREMIS with allied health professionals in Australia [21], combined with ongoing collaboration with Mexican physiotherapists working in mental health and violence-related fields. These disciplinary and geographical positions likely shaped the decision to frame the study around GBV, to use an IPV-derived instrument administered with broader GBV terminology, and to interpret the findings through both professional and health-system lenses. Data collection, ethics approvals, recruitment, and contextual interpretation were led by Latin American and Spanish collaborators, whose local professional knowledge was essential to the study’s feasibility and interpretation. We include this statement to acknowledge that the study design, framing, and interpretation were influenced by the positionalities, disciplinary backgrounds, and collaborative relationships of the research team. Authorship and roles were discussed explicitly among the core author team. While the senior author led protocol development, data analysis, and manuscript writing, first authorship was intentionally assigned to a locally based physiotherapist to reflect contextual leadership and contribution. Journal selection and dissemination strategy were jointly agreed, balancing institutional requirements, audience relevance, and the potential for impact within physiotherapy education and advocacy. Although structural asymmetries in academic publishing persist, this collaboration sought to minimise extractive practices through shared decision-making, transparency regarding roles, and a focus on generating evidence intended to support locally driven education, policy engagement, and professional advocacy. Making these processes explicit is intended to enhance transparency and accountability, rather than to claim their full resolution.

### Strengths and limitations

This study has several strengths. To our knowledge, it is the first multi-country assessment of physiotherapists’ preparedness to identify and respond to GBV conducted across Spanish-speaking contexts in Latin America and Spain. The use of the Spanish-language PREMIS instrument, originally validated in relation to IPV, provided a structured and comparable framework for assessment. The large sample size and broad geographic coverage across 15 countries provide novel insight into patterns of preparedness, knowledge, attitudes, and practice among physiotherapists working in diverse health-system contexts. In addition, collaboration with locally based physiotherapists and professional networks facilitated contextually grounded recruitment and interpretation of findings.

Several limitations should also be acknowledged. First, the sample was not nationally representative. Recruitment relied on voluntary participation through professional networks and online dissemination, and the proportion of physiotherapists captured varied substantially across countries. Findings should therefore be interpreted as indicative of patterns within this sample rather than as population-level prevalence estimates. Second, although the Spanish-language PREMIS provided a useful framework, it was originally developed in relation to IPV and was administered here with broader GBV framing. Some items, particularly within the objective knowledge domain, therefore remained more closely aligned with IPV than with the full spectrum of GBV. This measurement limitation should be considered when interpreting the findings.

Gender differences in preparedness and knowledge scores were observed, with male participants reporting slightly higher mean scores than female participants, and participants identifying as non-binary or another gender reporting higher scores. These findings should be interpreted cautiously, as gender was not a primary variable of interest, subgroup sizes were imbalanced, and the study was not designed or powered to examine gender-based differences. No inferential analyses were conducted to test these differences. Although the sample included participants from both Latin America and Spain, the study was not designed or powered to examine between-country or regional differences. Country-level subgroup sizes were highly variable and in some cases small, limiting the reliability of comparative analyses. In addition, recruitment relied on convenience sampling through professional networks, online dissemination, and, in some countries, organisational endorsement. This approach supported broad regional reach, but it may also have introduced selection bias towards physiotherapists who were more professionally connected, more engaged with online networks, or more interested in the study topic. Differences in organisational endorsement, network reach, and the extended recruitment period may also have contributed to variation in response patterns and unequal country-level representation. The primary aim was therefore to characterise overall preparedness across Spanish-speaking contexts rather than to conduct national comparisons. Finally, the cross-sectional, self-report design limits causal inference and may be subject to social desirability or recall bias. However, the consistency of findings across multiple PREMIS domains, including self-reported preparedness, perceived knowledge, opinions, and practice, supports the internal coherence of the results and strengthens confidence in the overall pattern observed.

## Conclusion

This multi-country study provides the first evidence on physiotherapists’ readiness to identify and respond to GBV across Spanish-speaking contexts in Latin America and Spain. In this sample, physiotherapists reported low levels of preparedness, limited training, infrequent identification of GBV in clinical practice, and substantial system-level barriers to response. These findings suggest a critical gap between the potential contribution of physiotherapy to health-sector responses to GBV and current clinical and organisational realities.

Future research should examine how GBV-related content can be embedded within physiotherapy education, continuing professional development, and service-level protocols, and should evaluate implementation strategies for supporting trauma-informed responses within routine physiotherapy practice.

## Supporting information

S1 Data De-identified minimal dataset required to replicate the reported findings. (CSV)

S2 Data Codebook for the de-identified minimal dataset, including variable names and coding. (CSV)

S1 Appendix Table A. Preparation sub-scale items. Table B. Perceived knowledge sub-scale items. Table C. Objective knowledge sub-scale items. Table D. Opinions sub-scale items. Table E. Practice sub-scale items: case identification, institutional resources, and protocols. Table F. Practice sub-scale items: frequency of GBV-related clinical actions. Table G. Practice sub-scale items: educational materials and referral resources. (DOCX)
