# Supplementary material for: Physiotherapists are underutilised in health-sector responses to gender-based violence: A cross-sectional survey of preparedness across Spanish-speaking countries
Source: PLOS Glob Public Health. 2026 Sep 2;6(9):e0007075. doi: 10.1371/journal.pgph.0007075 (PMC13537586; doi:10.1371/journal.pgph.0007075)
Supplement: Supplementary file 1 [file pgph.0007075.s004.pdf]

Physiotherapists are underutilised in health-sector responses to gender-based violence: a cross-sectional survey of preparedness across Spanish-speaking countries

Abstract

- Clarify issues regarding the use of the PREMIS instrument for broader GBV, rather than its original IPV focus. The abstract implies validated use of the instrument for GBV in general, but the manuscript later makes clear that the instrument remains partly IPV-framed.
- State that preparation and knowledge findings are self-reported where applicable.
- Indicate sampling approach.

Introduction

- The manuscript moves between GBV, IPV, and broader gendered violence constructs without sufficient conceptual precision. Distinguish these terms early in the introduction.
- The sentence beginning "GBV affects people across all genders..." contains a grammatical error.
- The presentation of LGBTQ+ populations is awkwardly phrased (rephrase).
- The manuscript promises coverage of all GBV populations and forms, yet the tool remains partly IPV-oriented.
- The research gap is relevant, but the final paragraph should explicitly state the aim or research questions.

Methods: overall design

- The design is identified as cross-sectional, but the section should specify the sampling strategy more explicitly, including whether this was convenience sampling.
- Provide the ethics approval reference number.
- The timeline is clear, but the manuscript should explain why recruitment extended over such a long period and whether this may have influenced response patterns.

Participants and recruitment

- Recruitment procedures need clearer methodological framing. The manuscript lists dissemination channels extensively, but does not adequately discuss selection bias, network effects, or unequal reach across countries.
- The variation between countries with formal organisational endorsement and countries relying on personal networks should be presented as a methodological limitation in this section, not only later.

- Clarify whether duplicate entries, ineligible responses, or incomplete surveys were screened out before analysis.
- The pretesting description is vague. State how many physiotherapists tested usability and whether any changes resulted.
- The section is descriptive in its naming of organisations; it should be condensed and focused on how recruitment was conducted.

Demographics

- The gender identity categories are too simplified. If "other" is used, explain how this was handled analytically and why more inclusive reporting categories were not retained in the main methods.
- The manuscript should explain how the lived experience of GBV variable was ethically handled analytically, not only in terms of participant protection.
- The wording around victim-survivor identification should be checked for consistency with the terminology used elsewhere in the paper.

Outcome measures

- The PREMIS is presented as validated, but the study applies it to GBV beyond IPV, which affects construct validity.
- The text says the instrument was both modified and not modified (It creates ambiguity).
- The manuscript should acknowledge that replacing IPV terminology with GBV in framing and instructions may alter how respondents interpret items.
- The assertion that the added LGBTQ+ response option did not alter the underlying construct is too strong, given the lack of psychometric testing in this sample.
- If one item was expanded, the manuscript should explain whether this affected comparability with prior PREMIS-based studies.
- Report reliability estimates for this sample rather than relying only on previous validation studies.
- The scoring explanation is not fully consistent across subscales. It should distinguish between perceived preparation, perceived knowledge, objective knowledge, opinions, and practice behaviours.

Data analysis

- Why was only descriptive analysis used despite the large sample and multiple subgroup variables?

- Pairwise deletion should be justified, especially given the substantial variation in denominators across analyses.
- Clearly report the extent and pattern of missingness.
- If no inferential testing was planned, that decision should be tied to the study aim in advance.

Results: participant characteristics

- The flow from 879 records to 860 consenting participants to 719 analytic cases is not sufficiently explained.
- The statement “All consenting participants were retained for analysis” is confusing because the analytic sample is smaller than the number of consenting participants.
- The handling of missing data is described repeatedly and redundantly.

Results: lived experience of GBV

- This section seems to be under-interpreted
- Clarify whether this variable was included as a descriptive characteristic only or whether it had any conceptual relevance to preparedness outcomes.
- Because subgroup numbers are very small for non-binary/other participants, the reporting risks of overinterpretation should be handled more cautiously.
- The term “lived experience” should be clearly defined (as it suggests that respondents were asked about lived experience).

Results: GBV training

- Distinguish between exposure, format, dose, and context more systematically.
- Passive exposure such as watching a video is grouped along training, which risks overstating educational exposure; define what counts as “formal training.”
- % are difficult to interpret without clearer denominators and category definitions.

Results: Preparation sub-scale

- “Preparedness” should be clarified as self-reported preparedness.
- The phrase “well or quite well prepared” should be explained in relation to the exact response categories.
- “~90%” should be replaced with exact figures where possible.

Results: Knowledge sub-scale

- There is an inconsistency between the methods, which describe an objective knowledge subscale, and the results, which repeatedly refer to perceived knowledge.
- Several knowledge items remain IPV-specific, reinforcing the need for greater caution in presenting this as a GBV knowledge measure.
- Report the overall objective knowledge score if that was indeed measured as described in the methods.

Results: Opinions sub-scale

- The reporting here is difficult to follow because domain means, item-level %, and interpretive statements are mixed together without a clear organising structure.
- The meaning of domain labels such as staff preparation, workplace issues, and staff constraints should be defined clearly in the methods or results sections.
- The item added for LGBTQ+ clients should be reported and interpreted carefully because it reduces direct comparability with the original measure.

Results: Practice sub-scale

- A distinction between case identification, clinical response, institutional resources, and educational materials is necessary
- The high proportion of "not applicable" responses requires careful handling because it complicates the interpretation of low practice engagement.
- It is unclear whether low reported action reflects non-identification, lack of opportunity, lack of competence, or system barriers.

Discussion

- Several claims are phrased too strongly, especially where the manuscript infers why physiotherapists do not respond to GBV despite not testing those mechanisms directly.
- The discussion repeats many results in detail instead of moving more decisively into interpretation.
- The manuscript overstates its methodological robustness by claiming that the use of the Spanish PREMIS demonstrates a broader gap in allied health readiness; this claim is weakened by the unresolved IPV-versus-GBV measurement issue.

Reflexivity and research partnership

- The section is long relative to the rest of the manuscript
- Much of the content is about authorship process and collaboration history rather than reflexivity in relation to data generation, interpretation, and positionality.
- The section should be shortened and focused more clearly on how researcher positions may have shaped the study.

Strengths and limitations

- The limitation regarding non-representativeness is important and should be brought forward more prominently.
- Country workforce comparisons are useful, but the basis for those estimates should be cited explicitly if retained.
- The measurement limitation regarding the adaptation from IPV to GBV should also be explicitly repeated here, not only in the discussion.

Conclusion

- The conclusion seems to adopt a strong, policy-oriented tone that goes beyond the descriptive nature of the findings.
- The final sentence appears incomplete and must be corrected.
