# Supplementary material for: Physiotherapists are underutilised in health-sector responses to gender-based violence: A cross-sectional survey of preparedness across Spanish-speaking countries
Source: PLOS Glob Public Health. 2026 Sep 2;6(9):e0007075. doi: 10.1371/journal.pgph.0007075 (PMC13537586; doi:10.1371/journal.pgph.0007075)
Supplement: Supplementary file 2 [file pgph.0007075.s006.pdf]

The authors have addressed most previous concerns. However, several key issues remain and must be resolved for the manuscript to be considered fully responsive.

- Clarify the use of the PREMIS instrument. PREMIS was designed to assess readiness for managing intimate partner violence. Since this study uses a wider GBV framework, the manuscript should not suggest that PREMIS is validated for all GBV types. Clearly state that the instrument was administered with a wider GBV framing, retaining some IPV-specific items. Ensure this clarification is consistent in the abstract, methods, discussion, and limitations sections.

- Describe the sampling approach in both the abstract and methods sections.

- The manuscript reports 879 imported records, 860 consenting participants, and 719 in the analytic sample. Please explain how these numbers are related and clarify whether records were excluded due to non-consent, ineligibility, duplication, incomplete surveys, or missing outcome data. The statement that all consenting participants were retained is unclear, given the smaller analytic sample.

- Clarify the distinction between perceived and objective knowledge. The methods describe an objective knowledge subscale, while some results refer to perceived knowledge. Clearly and consistently label self-reported preparedness, perceived knowledge, and objective knowledge throughout the manuscript.
