# Supplementary material for: Physiotherapists are underutilised in health-sector responses to gender-based violence: A cross-sectional survey of preparedness across Spanish-speaking countries
Source: PLOS Glob Public Health. 2026 Sep 2;6(9):e0007075. doi: 10.1371/journal.pgph.0007075 (PMC13537586; doi:10.1371/journal.pgph.0007075)
Supplement: Supplementary file 3 [file pgph.0007075.s003.docx]

Table A. Preparation Sub-Scale Items (n=604)

| Item, n (%) | Not prepared | Minimally | Slightly | Moderately | Fairly well | Well | Quite well |
| --- | --- | --- | --- | --- | --- | --- | --- |
| a. Ask appropriate questions about GBV | 104 (17) | 148 (24) | 131 (22) | 130 (22) | 37 (6) | 31 (5) | 23 (4) |
| b. Appropriately respond to disclosures of abuse | 76 (13) | 136 (22) | 155 (26) | 136 (22) | 47 (8) | 35 (6) | 19 (3) |
| c. Identify GBV indicators based on patient  history and physical examination | 93 (15) | 143 (24) | 141 (23) | 119 (20) | 50 (8) | 37 (6) | 21 (4) |
| d. Assess an GBV victim’s readiness to change | 164 (27) | 162 (27) | 120 (20) | 92 (15) | 29 (5) | 25 (4) | 11 (2) |
| e. Help an GBV victim assess his/her danger of lethality | 199 (33) | 151 (25) | 102 (17) | 80 (13) | 29 (5) | 24 (4) | 18 (3) |
| f. Conduct a safety assessment for the victim’s children | 255 (42) | 143 (24) | 85 (14) | 62 (10) | 27 (5) | 23 (4) | 9 (1) |
| g. Help an GBV victim create a safety plan | 238 (40) | 162 (27) | 80 (13) | 68 (11) | 25 (4) | 20 (3) | 11 (2) |
| h. Document GBV history and physical  examination findings in patient’s chart | 196 (33) | 156 (26) | 94 (16) | 89 (14) | 29 (5) | 24 (4) | 16 (2) |
| i. Make appropriate referrals for GBV | 222 (37) | 155 (26) | 94 (15) | 67 (11) | 31 (5) | 23 (4) | 12 (2) |
| j. Fulfil state reporting requirements for: |  |  |  |  |  |  |  |
| - GBV | 348 (58) | 109 (18) | 63 (10) | 39 (7) | 19 (3) | 14 (2) | 10 (2) |
| - Older adults | 338 (56) | 117 (19) | 68 (11) | 39 (7) | 19 (3) | 13 (2) | 9 (2) |
| - Children | 347 (58) | 110 (18) | 66 (10) | 37 (6) | 21 (4) | 13 (2) | 9 (2) |

Table B. Perceived Knowledge Sub-Scale Items (n=604)

| How much respondents feel they know about:  n, (%) | Not prepared | Minimally | Slightly | Moderately | Fairly well | Well | Quite well |
| --- | --- | --- | --- | --- | --- | --- | --- |
| Legal reporting requirements for: |  |  |  |  |  |  |  |
| - GBV | 174 (29) | 178 (30) | 110 (18) | 74 (12) | 28 (5) | 25 (4) | 13 (2) |
| - Older adults | 167 (28) | 190 (31) | 106 (18) | 70 (12) | 34 (5) | 23 (4) | 12 (2) |
| - Children | 172 (29) | 196 (32) | 102 (17) | 71 (12) | 33 (5) | 18 (3) | 10 (2) |
| Signs and symptoms of GBV | 91 (15) | 182 (30) | 142 (24) | 103 (17) | 54 (9) | 23 (4) | 8 (1) |
| How to document GBV in patient’s chart | 232 (38) | 157 (26) | 92 (15) | 64 (11) | 33 (6) | 18 (3) | 7 (1) |
| Referral sources for GBV victims | 237 (39) | 170 (28) | 93 (16) | 56 (9) | 28 (5) | 12 (2) | 6 (1) |
| Perpetrators of GBV | 223 (37) | 164 (27) | 110 (18) | 57 (10) | 26 (4) | 15 (3) | 6 (1) |
| Relationship between GBV and pregnancy | 238 (39) | 155 (26) | 100 (17) | 64 (11) | 21 (3) | 17 (3) | 6 (1) |
| Recognizing the childhood effects of witnessing GBV | 217 (36) | 164 (27) | 89 (15) | 69 (11) | 30 (5) | 24 (4) | 9 (2) |
| What questions to ask to identify GBV | 220 (37) | 159 (26) | 105 (18) | 68 (11) | 26 (4) | 18 (3) | 5 (1) |
| Why a victim might not disclose GBV | 152 (25) | 145 (24) | 119 (20) | 99 (16) | 39 (7) | 38 (6) | 10 (2) |
| Your role in detecting GBV | 187 (31) | 154 (25) | 114 (19) | 84 (14) | 39 (7) | 16 (3) | 8 (1) |
| What to say and not say in GBV situations with a patient | 174 (29) | 161 (27) | 112 (18) | 89 (15) | 37 (6) | 23 (4) | 7 (1) |
| Determining danger for a patient experiencing GBV | 196 (32) | 171 (28) | 100 (17) | 84 (14) | 29 (5) | 17 (3) | 6 (1) |
| Developing a safety plan with an GBV victim | 286 (47) | 139 (23) | 79 (13) | 51 (9) | 31 (5) | 10 (2) | 7 (1) |
| The stages an GBV victim experiences in understanding and changing his/her situation | 245 (41) | 147 (24) | 93 (15) | 68 (11) | 28 (5) | 12 (2) | 10 (2) |

Table C. Knowledge Sub-Scale Items (n=533)

| Item | Answers | n (%) correct |
| --- | --- | --- |
| 1. What is the most important risk factor for becoming a victim of GBV? | Female gender | 242 (46) |
| 2. Which is generally true about abusers? | They use violence as a means of controlling their partners | 315 (59) |
| 3. Which of the following are warning signs that a patient may have been abused by his/her partner? | a. Chronic pain without apparent cause | 300 (56) |
|  | b. Anxiety | 433 (81) |
|  | c. Substance abuse | 251 (47) |
|  | d. Frequent injuries | 421 (79) |
|  | e. Depression | 409 (77) |
| 4. Reasons why a victim may not be able to leave a violent relationship? | All six correct | 143 (27) |
|  | a. Fear of retaliation | 470 (88) |
|  | b. Financial dependence | 473 (89) |
|  | c. Religious beliefs | 318 (6) |
|  | d. Children’s needs | 419 (79) |
|  | e. Love for the partner | 211 (40) |
|  | f. Isolation | 286 (54) |
| 5. Most appropriate way to ask about GBV? | a. Are you a victim of GBV? (*incorrect*) | 77 (14) |
|  | b. Has your partner ever hurt or threatened you? (*correct)* | 130 (24) |
|  | c. Have you ever been afraid of your partner? (*incorrect*) | 483 (89) |
|  | d. Has your partner ever hit or hurt you? | 167 (31) |
| 6. Which of the following statements is generally true? | All five correct | 100 (19) |
|  | a. Many clients experiencing abuse show no visible injuries | 380 (71) |
|  | b. There are partner behaviour patterns that may indicate GBV | 403 (76) |
|  | c. Certain areas of the body are more frequently affected | 207 (39) |
|  | d. There are injury patterns commonly associated with GBV | 244 (46) |
|  | e. Injuries in different stages of healing may indicate abuse | 278 (52) |
| 7. Stage of change vignette | a. Precontemplation (denies there is a problem) | 369 (69) |
|  | b. Contemplation (Begins thinking abuse is not their own fault) | 300 (56) |
|  | c. Preparation (Begins making plans to leave abuser) | 275 (52) |
|  | d. Action (Obtains order(s) for protection) | 218 (41) |
|  | e. Maintenance (continues behaviour change) | 162 (30) |
|  |  |  |
| 8. True or False | a. Alcohol consumption is the greatest single predictor of the likelihood of GBV (*false*) | 175 (33) |
|  | b. There are no good reasons for not leaving an abusive relationship (*false*) | 102 (19) |
|  | c. Reasons for concern about GBV should not be included in a patient’s chart if s/he does not disclose the violence (*false)* | 339 (64) |
|  | d. When asking patients about IPV, physicians should use the words “abused” or “battered” (*false*) | 274 (51) |
|  | e. Being supportive of a patient’s choice to remain in a violent relationship would condone the abuse (*false)* | 333 (63) |
|  | f. Victims of GBV are able to make appropriate choices about how to handle their situation (t*rue)* | 115 (22) |
|  | g. Health care providers should not pressure patients to acknowledge that they are living in an abusive relationship (*true)* | 325 (61) |
|  | h. Victims of GBV are at greater risk of injury when they leave the relationship (*true)* | 271 (51) |
|  | i. Strangulation injuries are rare in cases of GBV (*false)* | 304 (57) |
|  | j. Allowing partners or friends to be present during a patient’s history and physical exam ensures safety for an IPV victim (*false)* | 331 (62) |

Table D. Opinions Sub-Scale Items (n=487)

|  | 1. Strongly disagree | 2. | 3. | 4. | 5. | 6. | 7. Strongly agree |
| --- | --- | --- | --- | --- | --- | --- | --- |
| 1. If a victim of GBV does not acknowledge the abuse, there is little I can do to help. | 99 (20) | 76 (16) | 112 (23) | 57 (12) | 70 (14) | 23 (5) | 50 (10) |
| 2. I ask every new client about the possibility of violence in their relationships. | 103 (21) | 64 (13) | 73 (15) | 102 (21) | 89 (18) | 32 (7) | 24 (5) |
| 3. The environment at my workplace encourages me to respond to GBV. | 66 (14) | 43 (9) | 54 (11) | 113 (23) | 123 (25) | 44 (9) | 44 (9) |
| 4. I can make appropriate referrals to community services for victims of GBV. | 44 (9) | 65 (13) | 65 (13) | 90 (18) | 130 (27) | 53 (11) | 40 (8) |
| 5. I am able to identify GBV without asking the client about it. | 60 (12) | 96 (20) | 103 (21) | 95 (20) | 99 (20) | 22 (5) | 12 (2) |
| 6. I do not have sufficient preparation to help clients deal with situations of GBV. | 22 (5) | 29 (6) | 41 (8) | 62 (13) | 124 (25) | 78 (16) | 131 (27) |
| 7. Patients who abuse alcohol or other drugs are likely to have a history of GBV. | 17 (4) | 23 (5) | 55 (11) | 104 (21) | 180 (37) | 63 (13) | 45 (9) |
| 8. Victims of abuse have the right to make their own decisions about whether health professionals should intervene. | 30 (6) | 33 (7) | 72 (15) | 100 (21) | 133 (27) | 54 (11) | 65 (13) |
| 9. I feel comfortable talking about GBV with my clients. | 30 (6) | 35 (7) | 86 (18) | 115 (24) | 129 (27) | 55 (11) | 36 (7) |
| 10. I do not have sufficient skills to talk about abuse with a female victim of GBV. | 27 (5) | 37 (8) | 93 (19) | 62 (13) | 133 (27) | 73 (15) | 62 (13) |
| 11. I do not have sufficient skills to talk about abuse with a male victim of GBV. | 30 (6) | 34 (7) | 73 (15) | 67 (14) | 140 (29) | 79 (16) | 64 (13) |
| 12. I do not have sufficient skills to talk about abuse with a victim of GBV from a different culture/ethnicity. | 29 (6) | 38 (8) | 60 (12) | 73 (15) | 136 (28) | 72 (15) | 78 (16) |
| 13. I do not have sufficient skills to talk about abuse with a victim of GBV from the LGBTQ+ community. | 35 (7) | 33 (7) | 74 (15) | 76 (16) | 124 (25) | 67 (14) | 77 (16) |
| 14. If a victim of GBV stays with their partner after repeated episodes of violence, they must accept responsibility for that violence. | 215 (44) | 70 (14) | 83 (17) | 52 (11) | 39 (8) | 14 (3) | 14 (3) |
| 15. I know the legal requirements for reporting suspected cases of GBV. | 137 (28) | 90 (19) | 106 (22) | 88 (18) | 42 (9) | 12 (2) | 12 (2) |
| 16. I know the legal requirements for reporting suspected cases of elder abuse. | 135 (28) | 87 (18) | 111 (23) | 75 (15) | 45 (9) | 15 (3) | 18 (4) |
| 17. I know the legal requirements for reporting suspected cases of child abuse. | 133 (27) | 93 (19) | 101 (21) | 76 (16) | 55 (11) | 14 (3) | 15 (3) |
| 18. Health professionals do not have time to support their clients regarding GBV. | 136 (28) | 64 (13) | 111 (23) | 72 (15) | 63 (13) | 24 (5) | 17 (3) |
| 19. I am able to gather the necessary information to identify GBV as the underlying cause of some conditions (e.g., depression, migraines). | 61 (13) | 69 (14) | 100 (21) | 92 (19) | 119 (24) | 31 (6) | 15 (3) |
| 20. If the client refuses to talk about the abuse, health professionals can only treat the physical injuries. | 62 (13) | 60 (12) | 140 (29) | 98 (20) | 94 (19) | 14 (3) | 19 (4) |
| 21. Victims of abuse could leave the relationship if they wanted to. | 93 (19) | 67 (14) | 81 (17) | 65 (13) | 87 (18) | 32 (6) | 62 (13) |
| 22. I comply with the regional/national protocol on GBV. | 105 (21) | 64 (13) | 81 (17) | 156 (32) | 50 (10) | 18 (4) | 13 (3) |
| 23. Health professionals have the responsibility to ask all clients about GBV | 36 (7) | 39 (8) | 57 (12) | 154 (32) | 132 (27) | 27 (6) | 41 (8) |
| 24. My workplace allows me adequate time to respond to victims of GBV. | 63 (13) | 67 (14) | 89 (18) | 100 (21) | 90 (19) | 35 (7) | 43 (8) |
| 25. I have contacted community services to establish referrals for GBV cases. | 157 (32) | 83 (17) | 82 (17) | 92 (19) | 43 (9) | 18 (4) | 10 (2) |
| 26. Alcohol abuse is a main cause of GBV. | 29 (6) | 31 (6) | 61 (13) | 117 (24) | 161 (33) | 50 (10) | 38 (8) |
| 27. Victims of abuse often have valid reasons to remain in an abusive relationship. | 61 (12) | 54 (11) | 92 (19) | 109 (22) | 115 (24) | 37 (8) | 19 (4) |
| 28. I am too busy to participate in a multidisciplinary team to manage GBV cases. | 117 (24) | 77 (16) | 128 (26) | 103 (21) | 48 (10) | 7 (1) | 7 (1) |
| 29. Screening for GBV will probably offend the people who are asked. | 45 (9) | 67 (14) | 111 (23) | 144 (30) | 89 (18) | 11 (2) | 20 (4) |
| 30. I have an adequate private space available to provide care to GBV victims. | 95 (20) | 82 (17) | 83 (17) | 82 (17) | 67 (14) | 29 (6) | 49 (10) |
| 31. I am able to gather the necessary information to identify GBV as the underlying cause of some injuries (e.g., bruises, fractures). | 61 (13) | 68 (14) | 72 (15) | 100 (20) | 123 (25) | 38 (8) | 24 (5) |
| 32. Women who move away from traditional roles are a main cause of GBV. | 134 (27) | 71 (15) | 86 (18) | 101 (21) | 65 (13) | 12 (2) | 18 (4) |
| 33. Health professionals do not have the knowledge to help clients regarding GBV. | 40 (8) | 32 (7) | 72 (15) | 108 (22) | 131 (27) | 54 (11) | 49 (10) |
| 34.I can adapt my therapeutic interventions depending on the stage the person with GBV is in. | 32 (7) | 29 (6) | 51 (11) | 104 (21) | 166 (34) | 55 (11) | 48 (10) |
| 35. I understand why victims of GBV do not always follow health professionals’ recommendations. | 20 (4) | 12 (2) | 40 (8) | 90 (19) | 190 (39) | 72 (15) | 62 (13) |
| 36. The use of alcohol and other drugs is related to GBV victimization. | 27 (6) | 28 (6) | 34 (7) | 137 (28) | 168 (34) | 42 (9) | 51 (10) |
| 37. I can recognize a victim of GBV by the way they behave. | 36 (7) | 50 (10) | 79 (16) | 129 (27) | 136 (28) | 27 (6) | 29 (6) |

Table E. Practice Sub-Scale Items (n=454)

| Item | Options | n (%) |  |
| --- | --- | --- | --- |
| 1. In the past 6 months, number of new GBV cases identified (acute, ongoing, or past disclosure) | None | 336 (74) |  |
|  | 1 – 5 | 74 (16) |  |
|  | 6 – 10 | 8 (2) |  |
|  | 11 – 20 | 3 (<1) |  |
|  | 21 or more | 3 (<1) |  |
|  | N/A – do not work in a clinic | 30 (6) |  |
|  |  |  |  |
| 2. In the last six months, which of the following actions have you taken when you identified a case of GBV? (Check all items that apply) | I have not identified GBV in the past 6 months | 346 (76) |  |
|  | I gave the client information (phone numbers, brochures, other information) | 28 (6) |  |
|  | I advised the client about the options they might have | 12 (3) |  |
|  | I conducted a risk assessment of the victim | 25 (6) |  |
|  | I conducted a risk assessment of the victim’s children | 74 (16) |  |
|  | I helped the client develop a safety plan | 39 (9) |  |
|  | I referred the client (to services/resources) | 56 (12) |  |
| Referral destination (among those who referred) | Individual therapy | 32 (7) |  |
|  | Couples therapy | 3 (1) |  |
|  | Child therapy / support group | 8 (2) |  |
|  | Primary care social worker | 16 (4) |  |
|  | Women’s program / shelters | 13 (3) |  |
|  | Alcohol / substance counselling | 6 (1) |  |
|  | Local GBV hotline | 13 (3) |  |
|  | Child protective services | 5 (1) |  |
|  | Legal support | 16 (4) |  |
|  | Perpetrator program | 2 (<1) |  |
|  | Religious organisation (church) | 4 (1) |  |
|  | Women’s GBV support group | 7 (2) |  |
|  | LGBTQ+ support group | 2 (<1) |  |
|  | National GBV hotline | 2(1) |  |
|  | Police / civil guard | 7 (2) |  |
|  | Housing/education/employment/financial support | 4 (1) |  |
|  | Other | 3 (1) |  |
|  |  |  |  |
| Is there a protocol for managing GBV cases in your service? | Yes, and it is widely used | 31 (7) |  |
|  | Yes, and it is used to some extent | 51 (11) |  |
|  | Yes, but it is not used | 20 (5) |  |
|  | No | 183 (40) |  |
|  | Not sure | 123 (27) |  |
|  | Not applicable to my clients | 26 (6) |  |
|  | Not in clinical practice | 20 (4) |  |
| Are you familiar with your institution’s policies and programs regarding the detection and management of GBV victims? | Yes | 89 (20) |  |
|  | No | 306 (67) |  |
|  | N/A | 58 (13) |  |
| Is a camera available to photograph injuries? | Yes | 78 (17) |  |
|  | No | 245 (54) |  |
|  | Not sure | 75 (17) |  |
|  | Not applicable to my clients | 31 (7) |  |
|  | Not in clinical practice | 22 (5) |  |
| Do you work in a jurisdiction where it is legally mandatory to report cases of GBV among adults? | Yes | 80 (17) |  |
|  | No | 150 (33) |  |
|  | Not sure | 180 (40) |  |
|  | N/A / not in clinical practice | 44 (10) |  |

Table F. Practice For each GBV victim you have identified in the past 6 months, how often have you carried out the following actions? (n=454)

| n (%) | Never | Rarely | Sometimes | Almost always | Always | N/A |
| --- | --- | --- | --- | --- | --- | --- |
| 1. Documented the client’s testimony in the medical record | 171 (38) | 31 (7) | 30 (7) | 18 (4) | 27 (6) | 173 (38) |
| 2. Used a body chart to document injuries | 197 (43) | 34 (8) | 21 (5) | 15 (3) | 7 (2) | 177 (39) |
| 3. Photographed injuries to include in the medical record | 212 (47) | 26 (6) | 16 (4) | 14 (3) | 7 (2) | 176 (39) |
| 4. Notified authorities when it was mandatory | 175 (39) | 27 (6) | 27 (6) | 21 (5) | 27 (6) | 173 (38) |
| 5. Conducted a risk assessment of the victim | 183 (40) | 32 (7) | 30 (7) | 16 (4) | 15 (3) | 175 (39) |
| 6. Conducted a risk assessment of the victim’s children | 189 (42) | 31 (7) | 24 (5) | 17 (4) | 13 (3) | 177 (39) |
| 7. Helped the client make a safety plan | 175 (39) | 31 (7) | 36 (8) | 19 (4) | 13 (3) | 177 (39) |
| 8. Contacted a service provider for GBV victims | 175 (39) | 27 (6) | 28 (6) | 21 (5) | 24 (6) | 173 (38) |
| 9. Offered validating/supportive communication | 135 (29) | 17 (4) | 36 (8) | 32 (7) | 61 (14) | 171 (38) |
| 10. Provided basic information about GBV | 151 (33) | 29 (7) | 44 (10) | 23 (5) | 31 (7) | 173 (38) |
| 11. Provided referral and information about other resources | 147 (32) | 33 (7) | 42 (9) | 23 (5) | 32 (7) | 174 (38) |

Table G. Practice questions (n=454)

|  |  | n (%) |
| --- | --- | --- |
| In your service, are educational materials and resources (leaflets, posters, information) related to GBV available? | Yes, clearly visible and accessible to clients | 34 (8) |
|  | Yes, but not clearly visible | 32 (7) |
|  | Yes, clearly visible, but not accessible to clients | 16 (4) |
|  | No | 263 (58) |
|  | Not applicable to my group of clients | 30 (7) |
|  | Not currently in clinical practice | 23 (5) |
|  | Not sure | 56 (12) |
| Do you provide educational materials related to GBV to your clients who are victims of violence? | Yes, almost always | 24 (5) |
|  | Yes, when it is safe for the client | 40 (9) |
|  | Yes, but only when the client asks for them | 30 (7) |
|  | No, because there are no adequate referral resources in the community | 145(32) |
|  | No, because I believe these materials are not generally useful | 10 (2) |
|  | No, other reason | 54 (12) |
|  | Not applicable to my group of clients | 108 (24) |
|  | I am not currently in clinical practice | 43 (9) |
| Do you feel you have appropriate referral resources for GBV cases in your community? | Yes | 41 (9) |
|  | No | 240 (53) |
|  | Not sure | 95 (21) |
|  | Not applicable to my group of clients | 40 (9) |
|  | I am not currently in clinical practice | 36 (8) |
|  |  |  |
|  |  |  |
|  |  |  |
|  |  |  |
