# Supplementary material for: Physiotherapists are underutilised in health-sector responses to gender-based violence: A cross-sectional survey of preparedness across Spanish-speaking countries
Source: PLOS Glob Public Health. 2026 Sep 2;6(9):e0007075. doi: 10.1371/journal.pgph.0007075 (PMC13537586; doi:10.1371/journal.pgph.0007075)
Supplement: Supplementary file 4 [file pgph.0007075.s007.docx]

| **No** | **Comment (Editor)** | **Response** |
| --- | --- | --- |
| 1 | Please amend your detailed Financial Disclosure statement. This is published with the article. It must therefore be completed in full sentences and contain the exact wording you wish to be published.  Please clarify all sources of financial support for your study. List the grants, grant numbers, and organizations that funded your study, including funding received from your institution. Please note that suppliers of material support, including research materials, should be recognized in the Acknowledgements section rather than in the Financial Disclosure.  ii. State the initials, alongside each funding source, of each author to receive each grant. For example: "This work was supported by the National Institutes of Health (####### to AM; ###### to CJ) and the National Science Foundation (###### to AM)."  iii. State what role the funders took in the study. If the funders had no role in your study, please state: “The funders had no role in study design, data collection and analysis, decision to publish, or preparation of the manuscript.”  iv. If any authors received a salary from any of your funders, please state which authors and which funders. | We have now amended this as requested |
| 2 | We note that your Data Availability Statement is currently as follows: All relevant data are within the paper and its Supporting Information files.  Please confirm at this time whether or not your submission contains all raw data required to replicate the results of your study. Authors must share the “minimal data set” for their submission. PLOS defines the minimal data set to consist of the data required to replicate all study findings reported in the article, as well as related metadata and methods (https://journals.plos.org/plosone/s/data-availability#loc-minimal-data-set-definition). | This has been addressed. We confirm that the revised submission includes the de-identified minimal dataset required to replicate the reported findings, together with a codebook describing variable names and coding, in the Supporting Information files. The Data Availability Statement has been updated accordingly.  *The de-identified minimal dataset required to replicate the findings reported in this study is provided in the Supporting Information files, together with a codebook describing variable names and coding.* |
| 3 | 3. Please ensure that your Ethics Statement is available in its entirety at the beginning of your Methods section, under a subheading 'Ethics Statement'. It must include:  i) The name(s) of the Institutional Review Board(s) or Ethics Committee(s)  ii) The approval number(s), or a statement that approval was granted by the named board(s)  iii) (for human participants or donors) - A statement that formal consent was obtained (must state whether verbal/written) OR the reason consent was not obtained (e.g., anonymity).  vi) (for child participants) - Please update your Ethics Statement with confirmation that written informed consent was obtained from the parent/guardian of each participant under 18 years of age. | This has been updated at the start of the Methods section and now reads:  *Ethics statement*  *Ethical approval for this study was obtained from the Research Committee of the Faculty of Health Sciences, Universidad Anáhuac México (approval reference 202269). All participants were aged 18 years or older and provided written informed consent electronically before commencing the online survey. Participation was voluntary and respondents could stop the survey at any time.* |
| 4 | 4. We notice that your supplementary material are included in the manuscript file. Please remove them and upload them with the file type 'Supporting Information'. Please ensure that each Supporting Information file has a legend listed in the manuscript after the references list. | This has been updated. |
|  | **Reviewer 1** | |
| 1 | Abstract:  Clarify issues regarding the use of the PREMIS instrument for broader GBV, rather than its original IPV focus. The abstract implies validated use of the instrument for GBV in general, but the manuscript later makes clear that the instrument remains partly IPV-framed.  • State that preparation and knowledge findings are self-reported where applicable.  • Indicate sampling approach. | We have revised the Abstract to clarify that the Spanish-language PREMIS was originally developed for intimate partner violence (IPV) and was administered in this study with broader gender-based violence (GBV) framing, rather than implying that it is a fully validated measure of GBV in all its forms. We have also clarified that the preparation and knowledge findings refer to self-reported preparedness and self-perceived knowledge where applicable. In addition, we now explicitly describe the study as a cross-sectional online convenience survey. |
| 2 | Introduction:  The manuscript moves between GBV, IPV, and broader gendered violence constructs without sufficient conceptual precision. Distinguish these terms early in the introduction. | We revised the opening paragraph of the Introduction to define gender-based violence (GBV) more clearly as an umbrella term and to position intimate partner violence (IPV) explicitly as one form of GBV. We also clarified that IPV is the most common form of violence against women globally.  The opening paragraph now reads:  *Gender-based violence (GBV) is a global public health and human-rights crisis. While GBV can affect anyone, it disproportionately affects women and girls (World Health Organization, 2021). GBV is an umbrella term that encompasses multiple forms of violence, including intimate partner violence (IPV), sexual violence, coercive and controlling behaviours, and violence directed at individuals because of their gender identity or sexual orientation (World Health Organization, 2013). The most common form of violence against women globally is IPV, and the World Health Organization (WHO) estimates that nearly one in three women worldwide has experienced physical and/or sexual violence in her lifetime, most commonly perpetrated by an intimate partner (Oram et al., 2022; World Health Organization, 2021).* |
| 3 | The sentence beginning “GBV affects people across all genders...” contains a grammatical error. | This has been updated and now reads:  *GBV also affects men and people of diverse sexual orientations and gender identities, including LGBTQ+ populations, often with substantial physical and psychological consequences that may be exacerbated by stigma related to identifying as a victim-survivor, minority stress, and barriers to help-seeking* |
| 4 | The presentation of LGBTQ+ populations is awkwardly phrased (rephrase). | This has been updated as above. |
| 5 | The manuscript promises coverage of all GBV populations and forms, yet the tool remains partly IPV-oriented. | This has been addressed by revising the Introduction to avoid implying that the study captures all forms of GBV equally. We now clarify that GBV is the broader umbrella term and acknowledge that the main available readiness instruments were originally developed in relation to IPV. More detailed clarification regarding the implications of this for interpretation has been added to the Methods and Discussion. |
| 6 | The research gap is relevant, but the final paragraph should explicitly state the aim or research questions. | This has been updated and now reads:  *Understanding physiotherapists’ readiness within Latin America and Spain is therefore a critical first step toward developing contextually relevant education, clinical protocols, and health-system strategies that strengthen the role of physiotherapy within comprehensive responses to GBV. Accordingly, this study aimed to describe Spanish-speaking physiotherapists’ training, self-reported preparedness, self-perceived knowledge, objective knowledge, opinions, and practice issues relating to GBV in Latin America and Spain.* |
| 7 | Methods: overall design  The design is identified as cross-sectional, but the section should specify the sampling strategy more explicitly, including whether this was convenience sampling. | This has been updated and now reads:  *This study used a cross-sectional, quantitative design and recruited a convenience sample of Spanish-speaking physiotherapists through an online survey. Recruitment began on November 15, 2024 and ended on August 15, 2025.* |
| 8 | Provide the ethics approval reference number. | The following has been added under sub-heading ‘Ethics statement’:  *Ethical approval for this study was obtained from the Research Committee of the Faculty of Health Sciences, Universidad Anáhuac México (approval reference 202269). All participants were aged 18 years or older and provided written informed consent electronically before commencing the online survey. Participation was voluntary and respondents could stop the survey at any time.* |
| 9 | The timeline is clear, but the manuscript should explain why recruitment extended over such a long period and whether this may have influenced response patterns. | This has now been addressed in two places. In the Methods, we added a brief operational explanation for the extended recruitment period. We also added a sentence to the limitations noting that the extended recruitment period and staged dissemination approach may have contributed to variation in response patterns across countries.  Methods  *Recruitment remained open from November 15, 2024 to August 15, 2025 to allow sufficient time to achieve an adequate sample size and to leverage dissemination opportunities across participating countries.*  Limitations  *In addition, recruitment relied on convenience sampling through professional networks, online dissemination, and, in some countries, organisational endorsement. This approach supported broad regional reach, but it may also have introduced selection bias towards physiotherapists who were more professionally connected, more engaged with online networks, or more interested in the study topic. Differences in organisational endorsement, network reach, and the extended recruitment period may also have contributed to variation in response patterns and unequal country-level representation.* |
| 10 | Participants and recruitment  Recruitment procedures need clearer methodological framing. The manuscript lists dissemination channels extensively, but does not adequately discuss selection bias, network  effects, or unequal reach across countries. | This has been addressed by revising the recruitment section to provide clearer methodological framing and to describe the sampling approach explicitly as convenience sampling. We also reduced the emphasis on listing individual dissemination channels and organisations, and instead clarified that recruitment occurred through professional networks, academic institutions, social media, and, in some countries, formal organisational support. To avoid overloading the Methods section with interpretive material, we now address the implications of this recruitment approach for selection bias, network effects, and unequal reach across countries in the Strengths and limitations section.  Methods  *Eligible participants were Spanish-speaking physiotherapists aged 18 years or older. Recruitment used convenience sampling and the survey was disseminated through professional physiotherapy networks, academic institutions, and social media platforms widely used in Latin America and Spain, including Facebook, Instagram, LinkedIn, and WhatsApp groups. The mode of dissemination varied across countries, with some recruitment supported by formal organisational endorsement and other recruitment relying primarily on personal and professional networks.*  Limitations  See response to Comment 9 above. |
| 11 | The variation between countries with formal organisational endorsement and countries relying on personal networks should be presented as a methodological limitation in this section, not only later. | This has been addressed by adding a sentence to the Participants and recruitment section to clarify that dissemination differed across countries. We have retained the fuller discussion of the implications of this for selection bias and unequal reach in the Strengths and limitations section.  It now reads:  *Recruitment used convenience sampling and the survey was disseminated through professional physiotherapy networks, academic institutions, and social media platforms widely used in Latin America and Spain, including Facebook, Instagram, LinkedIn, and WhatsApp groups. The mode of dissemination varied across countries, with some recruitment supported by formal organisational endorsement and other recruitment relying primarily on personal and professional networks.* |
| 12 | Clarify whether duplicate entries, ineligible responses, or incomplete surveys were screened out before analysis. | This has been clarified in the Participants and recruitment section. We now specify that responses were screened for consent status and completeness of demographic and survey data required for inclusion in the descriptive analyses, and we clarify that a separate formal duplicate-detection procedure was not undertaken.  It now reads:  *Responses were screened for consent status and completeness of demographic and survey data required for inclusion in the descriptive analyses. A separate formal duplicate-detection procedure was not undertaken.* |
| 13 | The pretesting description is vague. State how many physiotherapists tested usability and whether any changes resulted. | This has been clarified in the Methods and now reads:  *Usability and technical functionality were pretested with 11 physiotherapists prior to survey launch, and no substantive changes were required.* |
| 14 | The section is descriptive in its naming of organisations; it should be condensed and focused on how recruitment was conducted. | This has been addressed. See response to Comment 11 above. |
| 15 | Demographics:  The gender identity categories are too simplified. If “other” is used, explain how this was handled analytically and why more inclusive reporting categories were not retained in the main methods. | This has been addressed in two sections. The Methods has been updated and now reads:  *Demographic variables collected included age, gender identity (male, female, non-binary or other)*  Updated results now reads:  *Participants were predominantly female (73%, 26% male and 1% other), with a mean age of 35.3 years (SD 8.9, range 18–79). Given the very small numbers in the non-binary and other gender categories, these responses were combined for descriptive reporting and interpreted cautiously.* |
| 16 | The manuscript should explain how the lived experience of GBV variable was ethically handled analytically, not only in terms of participant protection. | This has been clarified in the Methods and Results. We now specify that the lived experience of GBV item was collected as an optional descriptive characteristic of the sample and was not used as a primary explanatory variable in the analysis.  Methods now read:  *To minimise potential distress, the question was presented with clear guidance about voluntary disclosure and was accompanied by links to national and international GBV support services available in Spanish. This variable was collected as an optional descriptive characteristic of the sample and was not used as a primary explanatory variable in the analysis.*  Results now read:  *More than one-third of respondents (n = 257, 36%) reported lived experience of GBV, while 34 (5%) preferred not to respond. This variable was included to describe the sample and was not analysed as a primary predictor of preparedness outcomes.* |
| 17 | The wording around victim-survivor identification should be checked for consistency with the terminology used elsewhere in the paper. | This has been checked and revised for consistency throughout the manuscript. We now use “victim-survivor” consistently when referring to people, while retaining “lived experience of GBV” when referring to the descriptive variable reported in the Results. |
| 18 | Outcome measures:  The PREMIS is presented as validated, but the study applies it to GBV beyond IPV, which affects construct validity. | This has been addressed by revising the Outcome Measures section to clarify that the Spanish-language PREMIS is a validated instrument originally developed in relation to intimate partner violence (IPV), and that in the present study it was administered with broader gender-based violence (GBV) framing. We now acknowledge explicitly that this affects interpretation, as PREMIS remains an IPV-derived instrument and some items may align more closely with IPV than with the full spectrum of GBV.  It now reads:  *The original instrument and the Spanish adaptation were developed and validated in relation to IPV. In the present study, PREMIS was administered with broader GBV framing to reflect the range of gendered violence that physiotherapists may encounter in clinical practice and consistent with previous research with allied health professionals (Wheatley et al., 2025). Participants were informed, in Spanish, that the term GBV would be used throughout the survey and was defined according to the United Nations (2023) as acts of harm directed against individuals or groups based on gender, including sexual, physical, psychological, and economic violence, occurring in both public and private settings. No modifications were made to the PREMIS items, response options, subscale structure, or scoring procedures; the adaptation was limited to the introductory framing and terminology. Replacing IPV terminology with broader GBV framing in the introductory instructions may also have influenced how some respondents interpreted individual items, particularly those that were originally developed in relation to partner violence. This approach improved alignment with the study aim, but the findings should be interpreted cautiously, as PREMIS remains an IPV-derived instrument and some items may align more closely with IPV than with the full spectrum of GBV (Russo & Pirlott, 2006; Wheatley et al., 2025).* |
| 19 | The text says the instrument was both modified and not modified (It creates ambiguity). | This has been clarified by revising the Outcome Measures section to distinguish between the validated Spanish-language PREMIS instrument and the broader GBV framing used when administering it in this study. We now make clear that no modifications were made to the PREMIS items, response options, subscale structure, or scoring procedures, and that the adaptation was limited to the introductory framing and terminology.  See response to Comment 18 above. |
| 20 | The manuscript should acknowledge that replacing IPV terminology with GBV in framing and instructions may alter how respondents interpret items. | This has now been acknowledged explicitly in the Outcome Measures section. We now state that replacing IPV terminology with broader GBV framing in the introductory instructions may have influenced how some respondents interpreted individual items, particularly those originally developed in relation to partner violence.  See response to Comment 18 above. |
| 21 | The assertion that the added LGBTQ+ response option did not alter the underlying construct is too strong, given the lack of psychometric testing in this sample. | This has been revised. We removed the assertion that the added LGBTQ+ response option did not alter the underlying construct. We now clarify that this additional response option was included as an exploratory descriptive addition and was not included in PREMIS domain scoring. This means it did not affect the scored PREMIS domain analyses, although the LGBTQ+ item itself should be interpreted descriptively.  It now reads:  This modification was introduced to better reflect the diversity of populations encountered in contemporary clinical practice and to explore potential differences in perceived self-efficacy across victim populations. This additional response option was reported descriptively and was not included in PREMIS domain scoring. Accordingly, comparability with prior PREMIS-based studies was retained for scored domain analyses, although the LGBTQ+ item itself should be interpreted as an exploratory descriptive addition. |
| 22 | If one item was expanded, the manuscript should explain whether this affected comparability with prior PREMIS-based studies. | This has now been clarified in the Outcome Measures section. We explain that the additional LGBTQ+ response option was included as an exploratory descriptive addition and was not included in PREMIS domain scoring. Accordingly, comparability with prior PREMIS-based studies was retained for scored domain analyses, although the LGBTQ+ item itself should be interpreted descriptively.  See updates to the manuscript reported in previous comment. |
| 23 | Report reliability estimates for this sample rather than relying only on previous validation  studies. | This has now been addressed by reporting reliability estimates for the relevant Likert-type multi-item scales in the present sample. We also clarify that internal consistency estimates were not emphasised for the objective knowledge and practice issues sections, as these comprise scored knowledge items and heterogeneous behavioural indicators rather than scales intended to function as internally consistent attitudinal measures.  The manuscript has been updated and now reads:  *In the present sample, internal consistency was high for the perceived preparation items (Cronbach’s α = 0.967) and perceived knowledge items (Cronbach’s α = 0.980). Reliability estimates were calculated for the Likert-type multi-item scales; the objective knowledge and practice issues sections comprised scored knowledge items and heterogeneous behavioural indicators, for which internal consistency estimates were not considered informative in the same way.* |
| 24 | The scoring explanation is not fully consistent across subscales. It should distinguish between perceived preparation, perceived knowledge, objective knowledge, opinions,  and practice behaviours. | This has been clarified by revising the scoring description to distinguish more explicitly between subscales. We now make clear that these were analysed separately, rather than treating “preparation” or “knowledge” as single undifferentiated constructs.  It now reads:  *The PREMIS includes conceptually distinct domains: perceived preparation, perceived knowledge, objective knowledge, opinions, and practice issues/behaviours. In this study, these were analysed separately to distinguish self-reported preparedness and knowledge from objective knowledge, attitudinal responses, and reported clinical practices. Summary scores were created for the relevant PREMIS components, with preparation and knowledge examined as distinct perceived and objective domains, alongside opinions and practice issues.*  And within the subscale descriptions:  *The Preparation section comprised two related but distinct self-report components: perceived preparation and perceived knowledge.*  *The Knowledge subscale assessed objective knowledge about GBV.*  *The Practice Issues subscale assessed self-reported practice behaviours and workplace practices related to GBV.* |
| 25 | Data analysis:  Why was only descriptive analysis used despite the large sample and multiple subgroup variables? | This has now been clarified in the Data analysis section. The study was designed as a descriptive cross-sectional survey, with the primary aim of characterising preparedness, knowledge, opinions, and practice relating to GBV among Spanish-speaking physiotherapists. Although the overall sample was large, the use of convenience sampling, variable subgroup sizes across countries and gender categories, and differing denominators across items and subscales meant that inferential subgroup comparisons were not considered appropriate for the aims of this study. We have therefore made it clearer that analyses were intentionally descriptive.  This has been updated and now reads:  *Consistent with the descriptive aim of the study, analyses focused on summarising levels of preparedness, knowledge, attitudes, and practice related to gender-based violence among physiotherapists across participating countries. Although the overall sample was large, the use of convenience sampling, highly variable subgroup sizes, and differing denominators across items and subscales meant that inferential subgroup comparisons were not considered appropriate. No inferential statistical testing was therefore undertaken, and results are presented descriptively to characterise patterns within the sample.* |
| 26 | Pairwise deletion should be justified, especially given the substantial variation in denominators across analyses. | This has now been clarified in the Data analysis section. We explain that pairwise deletion was used because the study was descriptive and we sought to retain as much available information as possible for each item and subscale, rather than excluding participants with partial missing data from all analyses. We also now state more clearly that this resulted in varying denominators across analyses.  This now reads:  *Pairwise deletion was used to retain as much available information as possible for each descriptive analysis, rather than excluding participants with partial missing data from all analyses.* |
| 27 | Clearly report the extent and pattern of missingness. | This has now been clarified in both the Data analysis and Results sections. We now report the extent and pattern of missingness more explicitly by noting that denominators varied across subscales and item-level analyses, and by providing the sample sizes contributing to each major PREMIS domain.  It now reads:  *Missingness was highest for some PREMIS subscales and item-level responses, resulting in varying denominators across analyses (e.g., n = 600 for perceived preparation, n = 597 for perceived knowledge, n = 533 for objective knowledge, n = 487 for opinions, and n = 454 for practice issues).* |
| 28 | If no inferential testing was planned, that decision should be tied to the study aim in advance. | This has been clarified in the Data analysis section. We now link the decision not to undertake inferential testing more explicitly to the descriptive aim of the study, rather than presenting it only as an analytic choice made after data collection.  It now reads:  *Consistent with the descriptive aim of the study, analyses focused on summarising levels of preparedness, knowledge, attitudes, and practice related to gender-based violence among physiotherapists across participating countries. Although the overall sample was large, the use of convenience sampling, highly variable subgroup sizes, and differing denominators across items and subscales meant that inferential subgroup comparisons were not considered appropriate. No inferential statistical testing was therefore undertaken, and results are presented descriptively to characterise patterns within the sample.* |
| 29 | Results: participant characteristics  The flow from 879 records to 860 consenting participants to 719 analytic cases is not sufficiently explained. | This has been clarified in the Results section. We revised the opening of the Participant Characteristics section to explain the flow from imported records to consenting participants to the final analytic sample more clearly, and to distinguish this from the later item-level missingness that contributed to varying denominators across analyses.  It now reads:  *Of the 879 records imported from the survey platform, 860 participants provided consent to take part. Records from participants who did not provide consent were excluded. The final analytic sample comprised 719 Spanish-speaking physiotherapists with sufficient demographic and outcome data for inclusion in the descriptive analyses. Missing responses were then retained and handled on an item-by-item basis, resulting in varying denominators across subscales and item-level analyses.* |
| 30 | The statement “All consenting participants were retained for analysis” is confusing because the analytic sample is smaller than the number of consenting participants. | This has been addressed as per response above. |
| 31 | The handling of missing data is described repeatedly and redundantly. | This has been addressed by streamlining the manuscript so that the handling of missing data is described primarily in the Data analysis section and briefly reflected in the Results through the reported denominators, rather than being repeated in multiple places. |
| 32 | Results: lived experience of GBV  This section seems to be under-interpreted | This has been addressed by adding brief descriptive interpretation to this section. We now note that lived experience of GBV was not uncommon within the surveyed physiotherapy workforce and that this provides important context for interpreting the sample, while also clarifying that subgroup patterns should be interpreted cautiously given the very small numbers in the non-binary and other gender categories.  It now reads:  *These findings indicate that lived experience of GBV was not uncommon within the surveyed physiotherapy workforce and provide important context for interpreting the sample… Given the very small numbers in the non-binary and other gender categories, these patterns should be interpreted cautiously.* |
| 33 | Clarify whether this variable was included as a descriptive characteristic only or whether it had any conceptual relevance to preparedness outcomes. | This has now been clarified. We specify that lived experience of GBV was included as a descriptive characteristic of the sample and was not analysed as a primary predictor of preparedness outcomes. |
| 34 | Because subgroup numbers are very small for non-binary/other participants, the reporting risks of overinterpretation should be handled more cautiously. | This has been addressed by adding a cautionary sentence to the Results section to avoid overinterpretation of the very small non-binary and other gender subgroups.  It now reads:  *Given the very small numbers in the non-binary and other gender categories, these patterns should be interpreted cautiously.* |
| 35 | The term “lived experience” should be clearly defined (as it suggests that respondents were asked about lived experience). | We now state more explicitly that the variable referred to as “lived experience of GBV” was based on respondents being asked whether they identified as a victim-survivor of any type of GBV.  It now reads:  *When asked whether they identified as a victim-survivor of any type of GBV, more than one-third of respondents (n = 257, 36%) answered yes…* |
| 36 | Results: GBV training  Distinguish between exposure, format, dose, and context more systematically. | This has been revised to distinguish more clearly between exposure, format, dose, and context of GBV training. We now report first how many respondents had no GBV training, then the types of GBV-related educational exposure reported, followed by the estimated number of training hours, and finally the setting in which training was received.  It now reads:  *Among the 719 respondents, nearly half (45%) reported having received no formal GBV training, defined here as structured education such as university-based teaching, basic courses, postgraduate training, or advanced/specialised training. Broader GBV-related educational exposure was also reported and included activities such as watching a video, reading a protocol or guideline, or attending a conference or awareness-raising session. The most commonly reported formats were watching a video (24%) or reading a protocol or guideline (19%), followed by attending a conference or awareness-raising session (18%). Less commonly reported formats included theoretical training during a university degree (11%), practical training during a university degree (4%), participation in a basic course of up to 20 hours (10%), or advanced/specialised training (2%). In terms of dose, when asked to estimate the total number of hours of training received (n=606), 15% reported less than 1 hour of training, 17% reported 1–3 hours, and 9% reported more than 10 hours. Training was most commonly received in the workplace (19%) or at university (18%), with a further 15% reporting informal or self-directed learning (e.g. social media or online platforms).* |
| 37 | Passive exposure such as watching a video is grouped along training, which risks overstating educational exposure; define what counts as “formal training.” | This has been updated as per response above. |
| 38 | % are difficult to interpret without clearer denominators and category definitions. | This has been addressed by clarifying category definitions and making the relevant denominators more explicit. In particular, we now state that the overall training-format percentages are based on the full sample, and we specify the denominator for the training-hours item, where missing data reduced the sample size.  It now reads:  *Among the 719 respondents, nearly half (45%) reported having received no formal GBV training… When asked to estimate the total number of hours of training received (n = 606), 15% reported less than 1 hour of training, 17% reported 1–3 hours, and 9% reported more than 10 hours.* |
| 39 | Results: Preparation sub-scale:  “Preparedness” should be clarified as self-reported preparedness. | This has been updated. |
| 40 | The phrase “well or quite well prepared” should be explained in relation to the exact response categories. | This has been clarified in the Results section. We now specify that the phrase “well or quite well prepared” refers to response categories 6 or 7 on the 7-point scale, and that the “well prepared” threshold refers to category 6 and above.  It now reads:  *…only 9–10% of respondents rated themselves as “well prepared” or “quite well prepared” (response categories 6 or 7 on the 7-point scale)…*  and:  *…between 90% and 96% of respondents rated themselves below the “well prepared” threshold (i.e., below response category 6).* |
| 41 | “~90%” should be replaced with exact figures where possible. | This has been revised in the Results section. We replaced the approximate expression “~90%” with more precise wording based on the item-level response distributions.  It now reads:  *Across all preparation items, between 90% and 96% of respondents rated themselves below the “well prepared” threshold.* |
| 42 | Results: knowledge sub-scale:  There is an inconsistency between the methods, which describe an objective knowledge subscale, and the results, which repeatedly refer to perceived knowledge. | This has been clarified in the Results section by distinguishing more explicitly between self-perceived knowledge and objective knowledge. We now report the mean perceived knowledge score separately and then describe the scored Knowledge sub-scale as objective knowledge.  It now reads:  *Self-perceived knowledge regarding GBV was low overall. The mean perceived knowledge score was 2.4 (SD = 1.27, n = 597) on the 7-point PREMIS scale… Objective knowledge was also limited and showed substantial variability across item-level domains (Supplementary Table S3).* |
| 43 | Several knowledge items remain IPV-specific, reinforcing the need for greater caution in presenting this as a GBV knowledge measure. | This has been addressed by revising the Results and Discussion to acknowledge more explicitly that several items within the objective knowledge section remain IPV-specific. We now use more cautious wording when interpreting this domain and avoid presenting it as a comprehensive measure of knowledge across the full spectrum of GBV.  It now reads:  *Objective knowledge was also limited and showed substantial variability across item-level domains (Supplementary Table S3). Several items within this section retained an IPV-specific framing, and the findings should therefore be interpreted cautiously as an IPV-derived indicator of GBV-related knowledge rather than a comprehensive measure of knowledge across all forms of GBV.* |
| 44 | Report the overall objective knowledge score if that was indeed measured as described in the methods. | This has been clarified by revising the Methods and Results to align more closely. Although the objective Knowledge section could be summarised as an overall scored total, we chose not to emphasise a single summary score in the revised manuscript because the section covers heterogeneous content and several items retain an IPV-specific framing. We therefore report objective knowledge primarily through item-level descriptive analyses, which we consider provides a more transparent account of the knowledge domains assessed. |
| 45 | Results: Opinion sub-scale  The reporting here is difficult to follow because domain means, item-level %, and interpretive statements are mixed together without a clear organising structure. | This has been addressed by revising the Opinions subsection to improve its organisation. We now present the domain-level scores first, followed by selected item-level findings, and we have reduced interpretive statements within the Results so that the section reads more clearly and descriptively.  It now reads, in part:  *At the domain level, mean scores ranged from 3.4 to 5.1 on the 7-point scale… At the item level, 43% of respondents agreed or strongly agreed that they lacked sufficient preparation to help clients experiencing GBV… Full item-level response distributions are presented in Supplementary Table S4.* |
| 46 | The meaning of domain labels such as staff preparation, workplace issues, and staff constraints should be defined clearly in the methods or results sections. | This has been clarified by defining the Opinions domain labels more explicitly in the Methods.  It now reads:  *The Opinions domains captured staff preparation (perceived readiness and role confidence), legal requirements (knowledge of reporting obligations), workplace issues (perceived organisational support and clinical environment), self-efficacy (confidence in identifying and responding to GBV), alcohol/drugs beliefs, victim understanding, victim autonomy, and staff constraints (perceived time and workload barriers).* |
| 47 | The item added for LGBTQ+ clients should be reported and interpreted carefully because it reduces direct comparability with the original measure. | This has been addressed as above. We now clarify that the additional LGBTQ+ response option was included as an exploratory descriptive addition and was not included in PREMIS domain scoring. |
| 48 | Results: Practice sub-scale  A distinction between case identification, clinical response, institutional resources,  and educational materials is necessary | This has been addressed and now reads, in part:  *Engagement with GBV-related practice was limited across four areas: case identification, clinical response following identification, institutional resources, and educational/referral materials (Supplementary Tables 5–7). Most physiotherapists reported not identifying any cases of GBV in the past six months (74%), with an additional 6% indicating that they did not work in a clinical setting. Among those who had identified at least one case, reported clinical responses such as referral, safety planning, and risk assessment were uncommon. Institutional systems to support GBV-related practice were also inconsistently reported, and availability and use of educational and referral materials was limited.* |
| 49 | The high proportion of “not applicable” responses requires careful handling because it complicates the interpretation of low practice engagement. | This has been clarified in the Results section. We now note that the high proportion of “not applicable” responses likely reflects both limited opportunity to apply these practices and the fact that some respondents were not currently working in clinical roles or in settings where particular practices were relevant. We also state more clearly that low reported engagement should therefore be interpreted cautiously.  It now reads:  *Across all items, a substantial proportion of respondents (38–39%) selected “not applicable.” This likely reflects both that many physiotherapists had not encountered situations in which these GBV-related practices could be applied during the reference period and that some respondents were not currently working in clinical roles or in settings where particular practices were relevant. Low reported engagement should therefore be interpreted cautiously and not assumed to reflect lack of action alone.* |
| 50 | It is unclear whether low reported action reflects non-identification, lack of opportunity, lack  of competence, or system barriers. | This has been clarified in the Results section. We now state more explicitly that low reported action may reflect a combination of non-identification, limited opportunity to apply these practices, low confidence or competence, and system-level barriers, rather than any single explanation.  It now reads:  *Accordingly, low reported action in this section may reflect a combination of non-identification, limited opportunity to apply these practices, low confidence or competence, and system-level barriers, rather than any single explanation.* |
| 51 | Discussion:  Several claims are phrased too strongly, especially where the manuscript infers why physiotherapists do not respond to GBV despite not testing those mechanisms directly. | This has been addressed through broader revisions to the Discussion. We have revised this section throughout to adopt more cautious wording, reduce repetition of detailed results, and avoid inferring mechanisms that were not directly tested in the present study. In particular, we have softened causal language and now frame possible explanations for low identification and response to GBV as interpretations suggested by the overall pattern of findings, rather than as conclusions established by the data. We have also moderated statements regarding the methodological strength of the study in light of the IPV-derived basis of PREMIS and the broader GBV framing used in this paper.  It now reads, for example:  *Taken together, the findings suggest that low identification and response to GBV in physiotherapy practice may be shaped by educational, organisational, and professional-role factors, although these mechanisms were not directly tested in the present study.*  And:  *Finally, by using the Spanish-language PREMIS instrument, originally validated in relation to IPV, this study extends existing allied health research into Spanish-speaking contexts. However, given the IPV-derived basis of the instrument and the broader GBV framing used here, the findings are best interpreted as evidence of an important readiness gap in this sample rather than as a definitive measure of allied health readiness across the full spectrum of GBV.* |
| 52 | The discussion repeats many results in detail instead of moving more decisively into interpretation. | This has been addressed through broader revision of the Discussion. We have reduced repetition of detailed results and revised the section to move more directly into interpretation and implications. |
| 53 | The manuscript overstates its methodological robustness by claiming that the use of the Spanish PREMIS demonstrates a broader gap in allied health readiness; this claim is  weakened by the unresolved IPV-versus-GBV measurement issue. | We now acknowledge more clearly that PREMIS is an IPV-derived instrument that was administered with broader GBV framing in this study, and we have tempered our interpretation accordingly.  It now reads:  *Finally, by using the Spanish-language PREMIS instrument, originally validated in relation to IPV, this study extends existing allied health research into Spanish-speaking contexts. However, given the IPV-derived basis of the instrument and the broader GBV framing used here, the findings are best interpreted as evidence of an important readiness gap in this sample rather than as a definitive measure of allied health readiness across the full spectrum of GBV.* |
| 54 | Reflexivity and research partnership:  The section is long relative to the rest of the manuscript. Much of the content is about authorship process and collaboration history rather than  reflexivity in relation to data generation, interpretation, and positionality.  The section should be shortened and focused more clearly on how researcher positions may have shaped the study. | This section has been substantially shortened and revised to focus more clearly on researcher positionality, data generation, and interpretation, while retaining a brief statement on authorship and collaboration because we consider this important to transparency in global health research partnerships. We removed much of the detailed collaboration history and reframed the section around how the disciplinary and geographical positions of the research team may have shaped study framing, instrument choice, recruitment, and interpretation.  It now reads, in part:  *These disciplinary and geographical positions likely shaped the decision to frame the study around GBV, to use an IPV-derived instrument administered with broader GBV terminology, and to interpret the findings through both professional and health-systems lenses. Data collection, ethics approvals, recruitment, and contextual interpretation were led by Latin American and Spanish collaborators, whose local professional knowledge was essential to the study’s feasibility and interpretation.*  We elected to retain the brief paragraph on collaboration and authorship. |
| 55 | Strengths and limitations:  The limitation regarding non-representativeness is important and should be brought forward more prominently. | This has been addressed by ensuring non-representativeness is identified more prominently as a primary limitation. We now state earlier and more directly that the sample was not nationally representative and that findings should be interpreted as indicative of patterns within this sample rather than as population-level estimates.  It now reads:  *Several limitations should also be acknowledged. First, the sample was not nationally representative. Recruitment relied on voluntary participation through professional networks and online dissemination, and the proportion of physiotherapists captured varied substantially across countries. Findings should therefore be interpreted as indicative of patterns within this sample rather than as population-level prevalence estimates.* |
| 56 | Country workforce comparisons are useful, but the basis for those estimates should be cited explicitly if retained. | This has been removed. |
| 57 | The measurement limitation regarding the adaptation from IPV to GBV should also be explicitly repeated here, not only in the discussion. | This has been addressed by explicitly repeating the measurement limitation in the Strengths and limitations section. We now state that, although the Spanish-language PREMIS provided a useful framework, it was originally developed in relation to IPV and was administered here with broader GBV framing, meaning that some items remained more closely aligned with IPV than with the full spectrum of GBV.  It now reads:  *Second, although the Spanish-language PREMIS provided a useful framework, it was originally developed in relation to IPV and was administered here with broader GBV framing. Some items, particularly within the objective knowledge domain, therefore remained more closely aligned with IPV than with the full spectrum of GBV. This measurement limitation should be considered when interpreting the findings.* |
| 58 | Conclusion:  The conclusion seems to adopt a strong, policy-oriented tone that goes beyond the descriptive nature of the findings.  The final sentence appears incomplete and must be corrected. | This has been revised, and now reads:  *This multi-country study provides the first evidence on physiotherapists’ readiness to identify and respond to GBV across Spanish-speaking contexts in Latin America and Spain. In this sample, physiotherapists reported low levels of preparedness, limited training, infrequent identification of GBV in clinical practice, and substantial system-level barriers to response. These findings suggest a critical gap between the potential contribution of physiotherapy to health-sector responses to GBV and current clinical and organisational realities. Future research should examine how GBV-related content can be embedded within physiotherapy education, continuing professional development, and service-level protocols, and should evaluate implementation strategies for supporting trauma-informed responses within routine physiotherapy practice.* |
|  |  |  |
|  | **Reviewer 2** | |
| 1 | This manuscript addresses an important and timely topic. The study has several notable strengths, including its large sample size, multi-country scope and use of a validated Spanish-language instrument. The findings are relevant to clinical practice, workforce development and health-profession education. Overall, the manuscript is well motivated and makes a meaningful contribution to an underexplored area.  At the same time, several points would benefit from clarification and revision to strengthen the manuscript’s conceptual framing, methodological transparency, and interpretation of results. | Many thanks for your constructive comments. |
| 2 | Major comments:  Clarify the conceptual relationship between GBV and IPV in the survey instrument. | This has been clarified throughout the manuscript. We now distinguish more explicitly between GBV as the broader umbrella construct and IPV as one form of GBV, and we clarify that the Spanish-language PREMIS was originally developed in relation to IPV but was administered in this study with broader GBV framing to reflect the range of gendered violence that physiotherapists may encounter in practice. We have also made clearer that this has implications for interpretation, as PREMIS remains an IPV-derived instrument and some items align more closely with IPV than with the full spectrum of GBV. |
| 3 | The manuscript states that the validated PREMIS instrument was adapted by replacing IPV with GBV in the introductory framing, while leaving the items and scoring procedures unchanged. Please clarify more explicitly how this adaptation affects interpretation of the results. Since the original PREMIS was designed for intimate partner violence, readers should understand whether the instrument is being used as a broader proxy for GBV readiness or whether it remains primarily an IPV-oriented measure with GBV terminology in the framing text. A brief justification of this adaptation would improve clarity. | This has been clarified more explicitly throughout the manuscript. We now state that the original PREMIS instrument and the Spanish adaptation were developed and validated in relation to IPV, and that in the present study the survey was administered with broader GBV framing to better reflect the range of gendered violence that physiotherapists may encounter in practice. We also now acknowledge more clearly that this affects interpretation: PREMIS remains an IPV-derived instrument, and some items align more closely with IPV than with the full spectrum of GBV. Accordingly, we interpret the findings as an IPV-derived indicator of readiness relevant to GBV practice, rather than as a definitive measure of readiness across all forms of GBV.  Updated text in the manuscript now reads:  *The original instrument and the Spanish adaptation were developed and validated in relation to IPV. In the present study, PREMIS was administered with broader GBV framing to reflect the range of gendered violence that physiotherapists may encounter in clinical practice. No modifications were made to the PREMIS items, response options, subscale structure, or scoring procedures; the adaptation was limited to the introductory framing and terminology.*  And:  *This approach improved alignment with the study aim, but the findings should be interpreted cautiously as PREMIS remains an IPV-derived instrument and that some items may align more closely with IPV than with the full spectrum of GBV.* |
| 4 | Strengthen the rationale for using GBV as the umbrella term.  The manuscript would benefit from a more explicit explanation of why GBV was selected as the broader construct, and how this choice aligns with the clinical realities of physiotherapy practice in Spanish-speaking settings. It would also be helpful to acknowledge that some items may be more directly applicable to IPV than to other forms of GBV. A short discussion of this limitation would make the interpretation more balanced. | These points have now been addressed throughout the manuscript. We clarify that PREMIS was originally developed and validated in relation to IPV but was administered in this study with broader GBV framing to better reflect the range of gendered violence that physiotherapists may encounter in practice. We also strengthened the rationale for using GBV as the umbrella term, while acknowledging more explicitly that some PREMIS items remain more directly applicable to IPV than to the broader spectrum of GBV. Accordingly, the findings are now interpreted more cautiously as an IPV-derived indicator of readiness relevant to GBV practice, rather than as a definitive measure of readiness across all forms of GBV.  It now reads, in part:  *The original instrument and the Spanish adaptation were developed and validated in relation to IPV. In the present study, PREMIS was administered with broader GBV framing to reflect the range of gendered violence that physiotherapists may encounter in clinical practice. No modifications were made to the PREMIS items, response options, subscale structure, or scoring procedures; the adaptation was limited to the introductory framing and terminology. This approach improved alignment with the study aim, but the findings should be interpreted in light of the fact that PREMIS remains an IPV-derived instrument and that some items may align more closely with IPV than with the full spectrum of GBV.* |
| 5 | Address the issue of representativeness more clearly.  The manuscript appropriately notes that the sample is not nationally representative. This limitation should be emphasised more strongly in the discussion, particularly because recruitment relied on online dissemination through professional networks and social media. Such a strategy is appropriate for reaching a wide regional sample, but it may also introduce selection bias toward more connected, engaged, or academically affiliated physiotherapists. Please make this limitation more explicit when interpreting the findings. | This has been addressed by strengthening the discussion of non-representativeness in the Strengths and limitations section. We now state more prominently that the sample was not nationally representative and clarify that recruitment through professional networks, online dissemination, and social media may have introduced selection bias towards physiotherapists who were more professionally connected, more engaged with online networks, or more interested in the study topic.  It now reads:  *First, the sample was not nationally representative. Recruitment relied on voluntary participation through professional networks and online dissemination, and the proportion of physiotherapists captured varied substantially across countries. Findings should therefore be interpreted as indicative of patterns within this sample rather than as population-level prevalence estimates.*  And later:  *In addition, recruitment relied on convenience sampling through professional networks, online dissemination, and, in some countries, organisational endorsement. This approach supported broad regional reach, but it may also have introduced selection bias towards physiotherapists who were more professionally connected, more engaged with online networks, or more interested in the study topic. Differences in organisational endorsement, network reach, and the extended recruitment period may also have contributed to variation in response patterns and unequal country-level representation.* |
| 6 | Provide a clearer account of missing data handling.  The methods state that analyses were conducted using all available data and that missing values were handled item-by-item. However, the results indicate different sample sizes across subscales and items. Please provide a clearer description of the extent of missing data for each major measure, and explain how many participants contributed to each subscale analysis. This will improve transparency and help readers assess the robustness of the descriptive findings. | This has been addressed by clarifying missing data handling in both the Data analysis and Results sections. We now state more explicitly that missing responses were handled on an item-by-item basis using pairwise deletion, resulting in varying denominators across analyses, and we report the sample sizes contributing to each major PREMIS domain.  It now reads in the Data analysis section:  *Analyses were conducted on all available data, with missing responses coded as missing and excluded on an item-by-item basis (pairwise deletion), resulting in varying denominators across subscales and items. Pairwise deletion was used to retain as much available information as possible for each descriptive analysis, rather than excluding participants with partial missing data from all analyses. The extent of missingness varied across measures and is reflected in the reported denominators for each subscale and item-level analysis.*  And in the Results section:  *Missing responses were then retained and handled on an item-by-item basis, resulting in varying denominators across subscales and item-level analyses.* |
| 7 | Ensure that interpretation remains descriptive.  Because the study is cross-sectional and no inferential subgroup comparisons were conducted, the discussion should remain clearly descriptive. Any differences noted by gender, country, or practice setting should be presented cautiously as observed patterns rather than interpreted as statistically meaningful differences. This will help maintain consistency between the methods and the interpretation of results. | This has been addressed through broader revision of the Discussion and Strengths and limitations sections. We have revised the interpretation to remain clearly descriptive and now present differences by gender, country, and practice setting more cautiously as observed patterns within the sample, rather than as statistically meaningful differences. We also make clearer that no inferential subgroup comparisons were undertaken.  It now reads, for example:  *Taken together, the findings suggest that low identification and response to GBV in physiotherapy practice may be shaped by educational, organisational, and professional-role factors, although these mechanisms were not directly tested in the present study.*  And in the limitations section:  *These findings should be interpreted cautiously, as gender was not a primary variable of interest, subgroup sizes were imbalanced, and the study was not designed or powered to examine gender-based differences. No inferential analyses were conducted to test these differences. Although the sample included participants from both Latin America and Spain, the study was not designed or powered to examine between-country or regional differences.* |
| 8 | Minor comments:  Please define GBV, IPV, and related terms consistently at first use and maintain the same terminology throughout the manuscript. | This has been addressed by revising the Introduction and related sections to define GBV and IPV more clearly at first use and to maintain this terminology more consistently throughout the manuscript. We now use GBV as the broader umbrella term, with IPV identified as one form of GBV, and we have aligned the wording across the Introduction, Outcome Measures, Discussion, and Strengths and limitations sections.  It now reads, in part:  *GBV is an umbrella term that encompasses multiple forms of violence, including intimate partner violence (IPV), sexual violence, coercive and controlling behaviours, and violence directed at individuals because of their gender identity or sexual orientation. The most common form of violence against women globally is IPV.* |
| 9 | A concise summary table of the PREMIS subscales, item numbers, and scoring approach would help readers understand the instrument more easily. | A concise summary table (Table 1) describing the PREMIS components, the construct assessed, item type, and scoring/reporting approach has been added. |
| 10 | Please ensure that country names, institutional names, and professional organisation names are formatted consistently throughout the manuscript. | This has been checked and revised throughout the manuscript. We have standardised the formatting of country names, institutional names, and professional organisation names for consistency across the main text, tables, and affiliations. |
| 11 | It would be helpful to add a short paragraph on how the findings could inform physiotherapy curriculum development, continuing professional education, and institutional protocols in Spanish-speaking contexts. | This has been addressed by revising the Discussion to include a clearer paragraph on the implications of the findings for physiotherapy education, continuing professional development, and institutional support. We now discuss the need for structured GBV-related content within undergraduate and postgraduate training, ongoing professional education, and clearer service-level protocols and referral pathways in Spanish-speaking contexts.  It now reads, in part:  *The findings of this study have clear implications for physiotherapy education and professional training… Therefore, embedding structured, skills-based GBV training within physiotherapy education may represent a critical opportunity to address this gap. Such training should extend beyond awareness-raising to include trauma-informed communication, recognition of health presentations associated with violence, clarification of professional roles and boundaries, and practical guidance on referral pathways and interprofessional collaboration.*  And later:  *Beyond individual knowledge and training gaps, the findings point to substantial system-level barriers that constrain physiotherapists’ ability to respond to GBV in practice… Strengthening system-level supports within health services and professional bodies may therefore be critical to translating improved education and training into sustained changes in clinical practice.* |
| 12 | Strengths of the study  The study addresses an important gap in the literature and provides valuable regional data from 15 Spanish-speaking countries. The large sample, broad geographic reach, and use of a validated survey instrument are important strengths. The manuscript also highlights a clinically relevant issue that has direct implications for training, policy, and survivor-centred care within physiotherapy. | We thank the reviewer for this positive assessment of the study’s strengths and contribution. |
| 13 | Overall recommendation  This is a strong and relevant manuscript that would benefit from revision to improve clarity, transparency, and interpretability. I would encourage the authors to address the points above, particularly those relating to instrument adaptation, representativeness, and missing-data reporting. With these revisions, the manuscript would make a useful contribution to the literature on GBV preparedness among physiotherapists. | We thank the reviewer for this constructive overall assessment. We have addressed the points raised regarding instrument adaptation, representativeness, and missing-data reporting, and we appreciate the reviewer’s recognition of the manuscript’s potential contribution to the literature on GBV preparedness among physiotherapists. |
