# Supplementary material for: Physiotherapists are underutilised in health-sector responses to gender-based violence: A cross-sectional survey of preparedness across Spanish-speaking countries
Source: PLOS Glob Public Health. 2026 Sep 2;6(9):e0007075. doi: 10.1371/journal.pgph.0007075 (PMC13537586; doi:10.1371/journal.pgph.0007075)
Supplement: Supplementary file 5 [file pgph.0007075.s008.docx]

| **No** | **Comment (Editor)** | **Response** |
| --- | --- | --- |
| 1 | We note that the ethics approval documents should not be published with the paper and are for internal information only. Please change the file type to 'Other' to ensure that they are not published. | This has been rectified. |
| 2 | We have noticed that you have uploaded Supporting Information files, but you have not included a list of legends. Please add a full list of legends for your Supporting Information files before or after the references list. | This has been updated. |
| 3 | If the reviewer comments include a recommendation to cite specific previously published works, please review and evaluate these publications to determine whether they are relevant and should be cited. There is no requirement to cite these works unless the editor has indicated otherwise. | N/A. |
|  | **Reviewer 1** | |
| 1 | Clarify the use of the PREMIS instrument. PREMIS was designed to assess readiness for managing intimate partner violence. Since this study uses a wider GBV framework, the manuscript should not suggest that PREMIS is validated for all GBV types. Clearly state that the instrument was administered with a wider GBV framing, retaining some IPV-specific items. Ensure this clarification is consistent in the abstract, methods, discussion, and limitations sections. | This has been clarified further throughout the manuscript. We now state consistently that PREMIS was originally developed and validated in relation to IPV, and that in the present study it was administered with broader GBV framing to reflect the range of gendered violence that physiotherapists may encounter in practice. We also make explicit that some items remained IPV-specific, particularly within the objective knowledge section, and that the findings should therefore be interpreted as deriving from an IPV-based instrument used with broader GBV framing. This clarification has been made consistently in the abstract, methods, discussion, and limitations sections.  Specific changes:  Abstract  *We conducted a cross-sectional online convenience survey of Spanish-speaking physiotherapists from Latin America and Spain using the Spanish-language Physician Readiness to Manage Intimate Partner Violence Survey (PREMIS), originally developed and validated for intimate partner violence (IPV) and administered here with broader GBV framing (violencia basada en género) while retaining some IPV-specific items.*  Introduction  *At the same time, the main available readiness instruments, including PREMIS were originally developed in relation to IPV. These instruments, they remain relevant to this question because IPV is one important form of GBV likely to be encountered in health care settings, although they may not fully capture readiness in relation to the broader spectrum of GBV.*  Methods  *In the present study, PREMIS was administered with broader GBV framing to reflect the range of gendered violence that physiotherapists may encounter in clinical practice, while retaining the original IPV-derived item structure, and consistent with previous research with allied health professionals (Wheatley et al., 2025).*  Discussion  *Interpretation of these findings should be considered in light of the measurement framework used. Although the PREMIS instrument was originally developed to assess readiness to manage intimate partner violence, the present study administered the Spanish-language version with broader GBV framing.*  Limitations  *The use of the validated Spanish-language PREMIS instrument, originally validated in relation to IPV, provided a structured and comparable framework for assessment.*  AND  *Second, although the Spanish-language PREMIS provided a useful framework, it was originally developed in relation to IPV and was administered here with broader GBV framing. Some items, particularly within the objective knowledge domain, therefore remained more closely aligned with IPV than with the full spectrum of GBV. This measurement limitation should be considered when interpreting the findings.* |
| 2 | Describe the sampling approach in both the abstract and methods sections. | We have ensured this is consistent.  Abstract  *We conducted a cross-sectional online convenience survey of Spanish-speaking physiotherapists from Latin America and Spain using the Spanish-language Physician Readiness to Manage Intimate Partner Violence Survey (PREMIS), originally developed and validated for intimate partner violence (IPV) and administered here with broader GBV framing (violencia basada en género) while retaining some IPV-specific items.*  Methods  *This study used a cross-sectional, online survey and recruited a convenience sample of Spanish-speaking physiotherapists. Recruitment began November 15, 2024 and ended August 15, 2025.* |
| 3 | The manuscript reports 879 imported records, 860 consenting participants, and 719 in the analytic sample. Please explain how these numbers are related and clarify whether records were excluded due to non-consent, ineligibility, duplication, incomplete surveys, or missing  outcome data. The statement that all consenting participants were retained is unclear, given the smaller analytic sample. | This has been clarified throughout the manuscript:  Methods  *Responses were screened for consent status and completeness of demographic and outcome data required for inclusion in the descriptive analyses. Records from participants who did not provide consent were excluded. A separate formal duplicate-detection procedure was not undertaken.*  Results  *Of the 879 records imported from the survey platform, 860 participants provided consent to take part. Records from participants who did not provide consent were excluded. The final analytic sample comprised 719 physiotherapists with complete demographic data and at least partial outcome data for inclusion in the descriptive analyses. Missing responses within the analytic sample were then retained and handled on an item-by-item basis, resulting in varying denominators across subscales and item-level analyses.* |
| 4 | Clarify the distinction between perceived and objective knowledge. The methods describe an objective knowledge subscale, while some results refer to perceived knowledge. Clearly and  consistently label self-reported preparedness, perceived knowledge, and objective knowledge throughout the manuscript. | This has been clarified further. We now distinguish explicitly between self-reported preparedness, perceived knowledge, and objective knowledge in both the Methods and Results sections.  Specific changes:  Methods  *Summary scores were created for the relevant PREMIS components, with self-reported preparation preparedness and perceived knowledge, and objective knowledge examined as distinct perceived and objective domains, alongside opinions and practice issues.*  AND  *Preparation Sub-Scale: The Preparation section comprised two related yet distinct self-report components: perceived preparation (e.g., ability to ask about GBV, make referrals, complete documentation) and perceived knowledge (e.g., legal obligations, signs and symptoms, referral resources). Responses were recorded on 7-point Likert scales (strongly disagree to strongly agree), with higher scores indicating greater self-reported preparedness or knowledge.*  *Knowledge Sub-Scale: The Knowledge section assessed objective knowledge about GBV/IPV-related content using multiple-choice, true/false, and stage-of-change items covering risk factors, warning signs, reasons survivors may remain in violent relationships, asking about violence, documentation, and legal/reporting issues.*  Results sub-headings changed to:  ‘Self-reported Preparedness Sub-Scale’ AND  ‘Perceived and objective Knowledge’ |
