# Supplementary material for: Physiotherapists are underutilised in health-sector responses to gender-based violence: A cross-sectional survey of preparedness across Spanish-speaking countries
Source: PLOS Glob Public Health. 2026 Sep 2;6(9):e0007075. doi: 10.1371/journal.pgph.0007075 (PMC13537586; doi:10.1371/journal.pgph.0007075)
Supplement: Supplementary file 6 [file pgph.0007075.s009.docx]

|  | **Reviewer 2** | |
| --- | --- | --- |
| 1 | Please perform a final proofreading of the manuscript to correct a small number of typographical and formatting errors. | We did a thorough proof-read and corrected minor typos. |
| 2 | For example:  "Sefl-reported preparedness" should be corrected to "Self-reported preparedness" (Results section) 402 line page-18. | This has been updated. |
| 3 | ",ore than one-third of respondents" should be corrected to "More than one-third of respondents" (Lived Experience of Gender-Based Violence section)363 line page-17. | This has been updated. |
| 4 | Minor spacing and punctuation inconsistencies should be checked throughout the manuscript. | This has been updated. |
| 5 | Although the handling of missing data has been explained, a brief statement summarizing participant flow and missing responses across major PREMIS domains would further improve transparency. | This has now been clarified further in the Results section. We added a brief summary sentence to distinguish participant flow from item-level missingness across the major PREMIS domains, in order to improve transparency regarding the relationship between imported records, consenting participants, the analytic sample, and varying denominators across subscales.  *“Thus, the difference between imported records, consenting participants, and the analytic sample reflects exclusion of non-consenting records and records with incomplete demographic data or insufficient outcome data, whereas variation across PREMIS denominators reflects item-level missing responses within the analytic sample.”* |
