# Supplementary material for: Physiotherapists are underutilised in health-sector responses to gender-based violence: A cross-sectional survey of preparedness across Spanish-speaking countries
Source: PLOS Glob Public Health. 2026 Sep 2;6(9):e0007075. doi: 10.1371/journal.pgph.0007075 (PMC13537586; doi:10.1371/journal.pgph.0007075)
Supplement: Peer review history

# Peer review: Decision Letter 0

**DOI:** 10.1371/journal.pgph.0007075.r001

**Authors:** Emma Campbell

13 Apr 2026

PGPH-D-26-00627

Physiotherapists are underutilised in health-sector responses to gender-based violence: a cross-sectional survey of preparedness across Spanish-speaking countries

PLOS Global Public Health

Dear Dr. Rosenbaum,

Thank you for submitting your manuscript to PLOS Global Public Health. After careful consideration, we feel that it has merit but does not fully meet PLOS Global Public Health’s publication criteria as it currently stands. Therefore, we invite you to submit a revised version of the manuscript that addresses the points raised during the review process.

The manuscript has been assessed by two reviewers and their comments are available below and in the attached document. Please review their comments and make the appropriate revisions to address any concerns they have raised.

Please submit your revised manuscript by May 28 2026 11:59PM. If you will need more time than this to complete your revisions, please reply to this message or contact the journal office at globalpubhealth@plos.org. When you're ready to submit your revision, log on to https://www.editorialmanager.com/pgph/ and select the 'Submissions Needing Revision' folder to locate your manuscript file.

Please include the following items when submitting your revised manuscript:

- A letter that responds to each point raised by the editor and reviewer(s). You should upload this letter as a separate file labeled 'Response to Reviewers'.
- A marked-up copy of your manuscript that highlights changes made to the original version. You should upload this as a separate file labeled 'Revised Manuscript with Track Changes'.
- An unmarked version of your revised paper without tracked changes. You should upload this as a separate file labeled 'Manuscript'.

Guidelines for resubmitting your figure files are available below the reviewer comments at the end of this letter.

As the corresponding author, your ORCID iD is verified in the submission system and will appear in the published article. PLOS supports the use of ORCID, and we encourage all coauthors to register for an ORCID iD and use it as well. Please encourage your coauthors to verify their ORCID iD within the submission system before final acceptance, as unverified ORCID iDs will not appear in the published article. *Only*  the individual author can complete the verification step; PLOS staff *cannot*  verify ORCID iDs on behalf of authors.

We look forward to receiving your revised manuscript.

Kind regards,

Emma Campbell, Ph.D

Staff Editor

PLOS Global Public Health

**Journal Requirements:**

1. Please amend your detailed Financial Disclosure statement. This is published with the article. It must therefore be completed in full sentences and contain the exact wording you wish to be published.

i. Please clarify all sources of financial support for your study. List the grants, grant numbers, and organizations that funded your study, including funding received from your institution. Please note that suppliers of material support, including research materials, should be recognized in the Acknowledgements section rather than in the Financial Disclosure.

ii. State the initials, alongside each funding source, of each author to receive each grant. For example: "This work was supported by the National Institutes of Health (####### to AM; ###### to CJ) and the National Science Foundation (###### to AM)."

iii. State what role the funders took in the study. If the funders had no role in your study, please state: “The funders had no role in study design, data collection and analysis, decision to publish, or preparation of the manuscript.”

iv. If any authors received a salary from any of your funders, please state which authors and which funders.

2. We note that your Data Availability Statement is currently as follows: All relevant data are within the paper and its Supporting Information files.

Please confirm at this time whether or not your submission contains all raw data required to replicate the results of your study. Authors must share the “minimal data set” for their submission. PLOS defines the minimal data set to consist of the data required to replicate all study findings reported in the article, as well as related metadata and methods (https://journals.plos.org/plosone/s/data-availability#loc-minimal-data-set-definition).

For example, authors should submit the following data:

- The values behind the means, standard deviations and other measures reported;

- The values used to build graphs;

- The points extracted from images for analysis.

Authors do not need to submit their entire data set if only a portion of the data was used in the reported study.

If your submission does not contain these data, please either upload them as Supporting Information files or deposit them to a stable, public repository and provide us with the relevant URLs, DOIs, or accession numbers. For a list of recommended repositories, please see https://journals.plos.org/plosone/s/recommended-repositories.

If there are ethical or legal restrictions on sharing a de-identified data set, please explain them in detail (e.g., data contain potentially sensitive information, data are owned by a third-party organization, etc.) and who has imposed them (e.g., an ethics committee). Please also provide contact information for a data access committee, ethics committee, or other institutional body to which data requests may be sent. If data are owned by a third party, please indicate how others may request data access.

3. Please ensure that your Ethics Statement is available in its entirety at the beginning of your Methods section, under a subheading 'Ethics Statement'. It must include:

i) The name(s) of the Institutional Review Board(s) or Ethics Committee(s)

ii) The approval number(s), or a statement that approval was granted by the named board(s)

iii) (for human participants or donors) - A statement that formal consent was obtained (must state whether verbal/written) OR the reason consent was not obtained (e.g., anonymity).

vi) (for child participants) - Please update your Ethics Statement with confirmation that written informed consent was obtained from the parent/guardian of each participant under 18 years of age.

4. We notice that your supplementary matrial are included in the manuscript file. Please remove them and upload them with the file type 'Supporting Information'. Please ensure that each Supporting Information file has a legend listed in the manuscript after the references list.

If the reviewer comments include a recommendation to cite specific previously published works, please review and evaluate these publications to determine whether they are relevant and should be cited. There is no requirement to cite these works unless the editor has indicated otherwise.

**Additional Editor Comments (if provided):**

[Note: HTML markup is below. Please do not edit.]

Reviewers' comments:

Reviewer's Responses to Questions

**Comments to the Author**

1. Does this manuscript meet PLOS Global Public Health’s publication criteria?

Reviewer #1: Partly

Reviewer #2: Yes

**********

2. Has the statistical analysis been performed appropriately and rigorously?

Reviewer #1: No

Reviewer #2: Yes

**********

3. Have the authors made all data underlying the findings in their manuscript fully available (please refer to the Data Availability Statement at the start of the manuscript PDF file)?

The PLOS Data policy

Reviewer #1: Yes

Reviewer #2: Yes

**********

4. Is the manuscript presented in an intelligible fashion and written in standard English?

Reviewer #1: Yes

Reviewer #2: Yes

**********

**Reviewer #1:**  Physiotherapists are underutilised in health-sector responses to gender-based violence: a cross-sectional survey of preparedness across Spanish-speaking countries

Abstract

• Clarify issues regarding the use of the PREMIS instrument for broader GBV, rather than its original IPV focus. The abstract implies validated use of the instrument for GBV in general, but the manuscript later makes clear that the instrument remains partly IPV-framed.

• State that preparation and knowledge findings are self-reported where applicable.

• Indicate sampling approach.

Introduction

• The manuscript moves between GBV, IPV, and broader gendered violence constructs without sufficient conceptual precision. Distinguish these terms early in the introduction.

• The sentence beginning “GBV affects people across all genders...” contains a grammatical error.

• The presentation of LGBTQ+ populations is awkwardly phrased (rephrase).

• The manuscript promises coverage of all GBV populations and forms, yet the tool remains partly IPV-oriented.

• The research gap is relevant, but the final paragraph should explicitly state the aim or research questions.

Methods: overall design

• The design is identified as cross-sectional, but the section should specify the sampling strategy more explicitly, including whether this was convenience sampling.

• Provide the ethics approval reference number.

• The timeline is clear, but the manuscript should explain why recruitment extended over such a long period and whether this may have influenced response patterns.

Participants and recruitment

• Recruitment procedures need clearer methodological framing. The manuscript lists dissemination channels extensively, but does not adequately discuss selection bias, network effects, or unequal reach across countries.

• The variation between countries with formal organisational endorsement and countries relying on personal networks should be presented as a methodological limitation in this section, not only later.

• Clarify whether duplicate entries, ineligible responses, or incomplete surveys were screened out before analysis.

• The pretesting description is vague. State how many physiotherapists tested usability and whether any changes resulted.

• The section is descriptive in its naming of organisations; it should be condensed and focused on how recruitment was conducted.

Demographics

• The gender identity categories are too simplified. If “other” is used, explain how this was handled analytically and why more inclusive reporting categories were not retained in the main methods.

• The manuscript should explain how the lived experience of GBV variable was ethically handled analytically, not only in terms of participant protection.

• The wording around victim-survivor identification should be checked for consistency with the terminology used elsewhere in the paper.

Outcome measures

• The PREMIS is presented as validated, but the study applies it to GBV beyond IPV, which affects construct validity.

• The text says the instrument was both modified and not modified (It creates ambiguity).

• The manuscript should acknowledge that replacing IPV terminology with GBV in framing and instructions may alter how respondents interpret items.

• The assertion that the added LGBTQ+ response option did not alter the underlying construct is too strong, given the lack of psychometric testing in this sample.

• If one item was expanded, the manuscript should explain whether this affected comparability with prior PREMIS-based studies.

• Report reliability estimates for this sample rather than relying only on previous validation studies.

• The scoring explanation is not fully consistent across subscales. It should distinguish between perceived preparation, perceived knowledge, objective knowledge, opinions, and practice behaviours.

Data analysis

• Why was only descriptive analysis used despite the large sample and multiple subgroup variables?

• Pairwise deletion should be justified, especially given the substantial variation in denominators across analyses.

• Clearly report the extent and pattern of missingness.

• If no inferential testing was planned, that decision should be tied to the study aim in advance.

Results: participant characteristics

• The flow from 879 records to 860 consenting participants to 719 analytic cases is not sufficiently explained.

• The statement “All consenting participants were retained for analysis” is confusing because the analytic sample is smaller than the number of consenting participants.

• The handling of missing data is described repeatedly and redundantly.

Results: lived experience of GBV

• This section seems to be under-interpreted

• Clarify whether this variable was included as a descriptive characteristic only or whether it had any conceptual relevance to preparedness outcomes.

• Because subgroup numbers are very small for non-binary/other participants, the reporting risks of overinterpretation should be handled more cautiously.

• The term “lived experience” should be clearly defined (as it suggests that respondents were asked about lived experience).

Results: GBV training

• Distinguish between exposure, format, dose, and context more systematically.

• Passive exposure such as watching a video is grouped along training, which risks overstating educational exposure; define what counts as “formal training.”

• % are difficult to interpret without clearer denominators and category definitions.

Results: Preparation sub-scale

• “Preparedness” should be clarified as self-reported preparedness.

• The phrase “well or quite well prepared” should be explained in relation to the exact response categories.

• “~90%” should be replaced with exact figures where possible.

Results: Knowledge sub-scale

• There is an inconsistency between the methods, which describe an objective knowledge subscale, and the results, which repeatedly refer to perceived knowledge.

• Several knowledge items remain IPV-specific, reinforcing the need for greater caution in presenting this as a GBV knowledge measure.

• Report the overall objective knowledge score if that was indeed measured as described in the methods.

Results: Opinions sub-scale

• The reporting here is difficult to follow because domain means, item-level %, and interpretive statements are mixed together without a clear organising structure.

• The meaning of domain labels such as staff preparation, workplace issues, and staff constraints should be defined clearly in the methods or results sections.

• The item added for LGBTQ+ clients should be reported and interpreted carefully because it reduces direct comparability with the original measure.

Results: Practice sub-scale

• A distinction between case identification, clinical response, institutional resources, and educational materials is necessary

• The high proportion of “not applicable” responses requires careful handling because it complicates the interpretation of low practice engagement.

• It is unclear whether low reported action reflects non-identification, lack of opportunity, lack of competence, or system barriers.

Discussion

• Several claims are phrased too strongly, especially where the manuscript infers why physiotherapists do not respond to GBV despite not testing those mechanisms directly.

• The discussion repeats many results in detail instead of moving more decisively into interpretation.

• The manuscript overstates its methodological robustness by claiming that the use of the Spanish PREMIS demonstrates a broader gap in allied health readiness; this claim is weakened by the unresolved IPV-versus-GBV measurement issue.

Reflexivity and research partnership

• The section is long relative to the rest of the manuscript

• Much of the content is about authorship process and collaboration history rather than reflexivity in relation to data generation, interpretation, and positionality.

• The section should be shortened and focused more clearly on how researcher positions may have shaped the study.

Strengths and limitations

• The limitation regarding non-representativeness is important and should be brought forward more prominently.

• Country workforce comparisons are useful, but the basis for those estimates should be cited explicitly if retained.

• The measurement limitation regarding the adaptation from IPV to GBV should also be explicitly repeated here, not only in the discussion.

Conclusion

• The conclusion seems to adopt a strong, policy-oriented tone that goes beyond the descriptive nature of the findings.

• The final sentence appears incomplete and must be corrected.

**Reviewer #2:**  This manuscript addresses an important and timely topic. The study has several notable strengths, including its large sample size, multi-country scope and use of a validated Spanish-language instrument. The findings are relevant to clinical practice, workforce development and health-profession education. Overall, the manuscript is well motivated and makes a meaningful contribution to an underexplored area.

At the same time, several points would benefit from clarification and revision to strengthen the manuscript’s conceptual framing, methodological transparency, and interpretation of results.

Major comments

Clarify the conceptual relationship between GBV and IPV in the survey instrument.

The manuscript states that the validated PREMIS instrument was adapted by replacing IPV with GBV in the introductory framing, while leaving the items and scoring procedures unchanged. Please clarify more explicitly how this adaptation affects interpretation of the results. Since the original PREMIS was designed for intimate partner violence, readers should understand whether the instrument is being used as a broader proxy for GBV readiness or whether it remains primarily an IPV-oriented measure with GBV terminology in the framing text. A brief justification of this adaptation would improve clarity.

Strengthen the rationale for using GBV as the umbrella term.

The manuscript would benefit from a more explicit explanation of why GBV was selected as the broader construct, and how this choice aligns with the clinical realities of physiotherapy practice in Spanish-speaking settings. It would also be helpful to acknowledge that some items may be more directly applicable to IPV than to other forms of GBV. A short discussion of this limitation would make the interpretation more balanced.

Address the issue of representativeness more clearly.

The manuscript appropriately notes that the sample is not nationally representative. This limitation should be emphasised more strongly in the discussion, particularly because recruitment relied on online dissemination through professional networks and social media. Such a strategy is appropriate for reaching a wide regional sample, but it may also introduce selection bias toward more connected, engaged, or academically affiliated physiotherapists. Please make this limitation more explicit when interpreting the findings.

Provide a clearer account of missing data handling.

The methods state that analyses were conducted using all available data and that missing values were handled item-by-item. However, the results indicate different sample sizes across subscales and items. Please provide a clearer description of the extent of missing data for each major measure, and explain how many participants contributed to each subscale analysis. This will improve transparency and help readers assess the robustness of the descriptive findings.

Ensure that interpretation remains descriptive.

Because the study is cross-sectional and no inferential subgroup comparisons were conducted, the discussion should remain clearly descriptive. Any differences noted by gender, country, or practice setting should be presented cautiously as observed patterns rather than interpreted as statistically meaningful differences. This will help maintain consistency between the methods and the interpretation of results.

Minor comments

Please define GBV, IPV, and related terms consistently at first use and maintain the same terminology throughout the manuscript.

A concise summary table of the PREMIS subscales, item numbers, and scoring approach would help readers understand the instrument more easily.

The introduction and discussion both contain substantial background on the public-health burden of GBV. Consider trimming repeated material to improve flow and readability.

Please ensure that country names, institutional names, and professional organisation names are formatted consistently throughout the manuscript.

It would be helpful to add a short paragraph on how the findings could inform physiotherapy curriculum development, continuing professional education, and institutional protocols in Spanish-speaking contexts.

Strengths of the study

The study addresses an important gap in the literature and provides valuable regional data from 15 Spanish-speaking countries. The large sample, broad geographic reach, and use of a validated survey instrument are important strengths. The manuscript also highlights a clinically relevant issue that has direct implications for training, policy, and survivor-centred care within physiotherapy.

Overall recommendation

This is a strong and relevant manuscript that would benefit from revision to improve clarity, transparency, and interpretability. I would encourage the authors to address the points above, particularly those relating to instrument adaptation, representativeness, and missing-data reporting. With these revisions, the manuscript would make a useful contribution to the literature on GBV preparedness among physiotherapists.

**********

what does this mean?). If published, this will include your full peer review and any attached files.

**Do you want your identity to be public for this peer review?**  If you choose “no”, your identity will remain anonymous but your review may still be made public.

For information about this choice, including consent withdrawal, please see our Privacy Policy

Reviewer #1: **Yes:**  Desmond Mathye

Reviewer #2: **Yes:**  Dr. Smati Sambyal PT

**********

[NOTE: If reviewer comments were submitted as an attachment file, they will be attached to this email and accessible via the submission site. Please log into your account, locate the manuscript record, and check for the action link "View Attachments". If this link does not appear, there are no attachment files.]

**Figure Resubmissions:**

Attachment Submitted filename: PLOS 1 Comments.pdf

# Author comment: Author response to Decision Letter 1

**DOI:** 10.1371/journal.pgph.0007075.r002

4 May 2026

Attachment Submitted filename: Response to reviewers.docx

# Peer review: Decision Letter 1

**DOI:** 10.1371/journal.pgph.0007075.r003

**Authors:** Johanna Pruller

31 May 2026

PGPH-D-26-00627R1

Physiotherapists are underutilised in health-sector responses to gender-based violence: a cross-sectional survey of preparedness across Spanish-speaking countries

PLOS Global Public Health

Dear Dr. Rosenbaum,

Thank you for submitting your manuscript to PLOS Global Public Health. After careful consideration, we feel that it has merit but does not fully meet PLOS Global Public Health’s publication criteria as it currently stands. Therefore, we invite you to submit a revised version of the manuscript that addresses the points raised during the review process.

==============================

The manuscript has been evaluated by two reviewers, and their comments are available below.

The reviewers have raised a number of concerns that need attention. Could you please revise the manuscript to carefully address the concerns raised?

==============================

Please submit your revised manuscript by Jul 28 2026 11:59PM. If you will need more time than this to complete your revisions, please reply to this message or contact the journal office at globalpubhealth@plos.org. When you're ready to submit your revision, log on to https://www.editorialmanager.com/pgph/ and select the 'Submissions Needing Revision' folder to locate your manuscript file.

Please include the following items when submitting your revised manuscript:

- A letter that responds to each point raised by the editor and reviewer(s). You should upload this letter as a separate file labeled 'Response to Reviewers'.
- A marked-up copy of your manuscript that highlights changes made to the original version. You should upload this as a separate file labeled 'Revised Manuscript with Track Changes'.
- An unmarked version of your revised paper without tracked changes. You should upload this as a separate file labeled 'Manuscript'.

Guidelines for resubmitting your figure files are available below the reviewer comments at the end of this letter.

As the corresponding author, your ORCID iD is verified in the submission system and will appear in the published article. PLOS supports the use of ORCID, and we encourage all coauthors to register for an ORCID iD and use it as well. Please encourage your coauthors to verify their ORCID iD within the submission system before final acceptance, as unverified ORCID iDs will not appear in the published article. *Only*  the individual author can complete the verification step; PLOS staff *cannot*  verify ORCID iDs on behalf of authors.

We look forward to receiving your revised manuscript.

Kind regards,

Johanna Pruller, Ph.D.

PLOS Staff Editor

PLOS Global Public Health

Journal Requirements:

1. We note that the ethics approval documents should not be published with the paper and are for internal information only. Please change the file type to 'Other' to ensure that they are not published.

2. We have noticed that you have uploaded Supporting Information files, but you have not included a list of legends. Please add a full list of legends for your Supporting Information files before or after the references list.

3. If the reviewer comments include a recommendation to cite specific previously published works, please review and evaluate these publications to determine whether they are relevant and should be cited. There is no requirement to cite these works unless the editor has indicated otherwise.

Additional Editor Comments (if provided):

[Note: HTML markup is below. Please do not edit.]

Reviewers' comments:

Reviewer's Responses to Questions

**Comments to the Author**

Reviewer #1: All comments have been addressed

Reviewer #2: All comments have been addressed

**********

publication criteria?

Reviewer #1: Yes

Reviewer #2: Yes

**********

3. Has the statistical analysis been performed appropriately and rigorously?

Reviewer #1: Yes

Reviewer #2: Yes

**********

4. Have the authors made all data underlying the findings in their manuscript fully available (please refer to the Data Availability Statement at the start of the manuscript PDF file)?

The PLOS Data policy

Reviewer #1: Yes

Reviewer #2: Yes

**********

5. Is the manuscript presented in an intelligible fashion and written in standard English?

Reviewer #1: Yes

Reviewer #2: Yes

**********

Reviewer #1: • Clarify the use of the PREMIS instrument. PREMIS was designed to assess readiness for managing intimate partner violence. Since this study uses a wider GBV framework, the manuscript should not suggest that PREMIS is validated for all GBV types. Clearly state that the instrument was administered with a wider GBV framing, retaining some IPV-specific items. Ensure this clarification is consistent in the abstract, methods, discussion, and limitations sections.

• Describe the sampling approach in both the abstract and methods sections.

• The manuscript reports 879 imported records, 860 consenting participants, and 719 in the analytic sample. Please explain how these numbers are related and clarify whether records were excluded due to non-consent, ineligibility, duplication, incomplete surveys, or missing outcome data. The statement that all consenting participants were retained is unclear, given the smaller analytic sample.

• Clarify the distinction between perceived and objective knowledge. The methods describe an objective knowledge subscale, while some results refer to perceived knowledge. Clearly and consistently label self-reported preparedness, perceived knowledge, and objective knowledge throughout the manuscript.

Reviewer #2: The authors have addressed the previously raised comments satisfactorily. The revisions have improved clarity, particularly regarding the adaptation of the PREMIS instrument, the rationale for using GBV as the umbrella construct, and the discussion of sampling limitations and missing data handling. The interpretation is now appropriately aligned with the descriptive nature of the study.

I have no further major concerns and recommend acceptance in its current form.

**********

what does this mean?). If published, this will include your full peer review and any attached files.

**Do you want your identity to be public for this peer review?**  If you choose “no”, your identity will remain anonymous but your review may still be made public.

For information about this choice, including consent withdrawal, please see our Privacy Policy

Reviewer #1: **Yes:**  Desmond Mathye

Reviewer #2: **Yes:**  Smati Sambyal

**********

[NOTE: If reviewer comments were submitted as an attachment file, they will be attached to this email and accessible via the submission site. Please log into your account, locate the manuscript record, and check for the action link "View Attachments". If this link does not appear, there are no attachment files.]

Attachment Submitted filename: PLOS 1 Reviewers Comment May 2026.pdf

# Author comment: Author response to Decision Letter 2

**DOI:** 10.1371/journal.pgph.0007075.r004

1 Jun 2026

Attachment Submitted filename: Response to reviewers 2.docx

# Peer review: Decision Letter 2

**DOI:** 10.1371/journal.pgph.0007075.r005

**Authors:** Jennifer Tucker

22 Jun 2026

PGPH-D-26-00627R2

Physiotherapists are underutilised in health-sector responses to gender-based violence: a cross-sectional survey of preparedness across Spanish-speaking countries

PLOS Global Public Health

Dear Dr. Rosenbaum,

Thank you for submitting your manuscript to PLOS Global Public Health. After careful consideration, we feel that it has merit but does not fully meet PLOS Global Public Health’s publication criteria as it currently stands. Therefore, we invite you to submit a revised version of the manuscript that addresses the points raised during the review process.

A few minor revisions have been requested before this can proceed to publication. Please address all reviewer comments below, with particular attention to copyediting, explanation of participant flow and missing responses.

Please submit your revised manuscript by Jul 19 2026 11:59PM. If you will need more time than this to complete your revisions, please reply to this message or contact the journal office at globalpubhealth@plos.org. When you're ready to submit your revision, log on to https://www.editorialmanager.com/pgph/ and select the 'Submissions Needing Revision' folder to locate your manuscript file.

Please include the following items when submitting your revised manuscript:

- A letter that responds to each point raised by the editor and reviewer(s). You should upload this letter as a separate file labeled 'Response to Reviewers'.
- A marked-up copy of your manuscript that highlights changes made to the original version. You should upload this as a separate file labeled 'Revised Manuscript with Track Changes'.
- An unmarked version of your revised paper without tracked changes. You should upload this as a separate file labeled 'Manuscript'.

Guidelines for resubmitting your figure files are available below the reviewer comments at the end of this letter.

As the corresponding author, your ORCID iD is verified in the submission system and will appear in the published article. PLOS supports the use of ORCID, and we encourage all coauthors to register for an ORCID iD and use it as well. Please encourage your coauthors to verify their ORCID iD within the submission system before final acceptance, as unverified ORCID iDs will not appear in the published article. *Only*  the individual author can complete the verification step; PLOS staff *cannot*  verify ORCID iDs on behalf of authors.

We look forward to receiving your revised manuscript.

Kind regards,

Jennifer Tucker, PhD

Staff Editor

PLOS Global Public Health

Journal Requirements:

If the reviewer comments include a recommendation to cite specific previously published works, please review and evaluate these publications to determine whether they are relevant and should be cited. There is no requirement to cite these works unless the editor has indicated otherwise.

Please review your reference list to ensure that it is complete and correct. If you have cited papers that have been retracted, please include the rationale for doing so in the manuscript text, or remove these references and replace them with relevant current references. Any changes to the reference list should be mentioned in the rebuttal letter that accompanies your revised manuscript. If you need to cite a retracted article, indicate the article’s retracted status in the References list and also include a citation and full reference for the retraction notice.

Additional Editor Comments (if provided):

[Note: HTML markup is below. Please do not edit.]

Reviewers' comments:

Reviewer's Responses to Questions

**Comments to the Author**

Reviewer #1: All comments have been addressed

Reviewer #2: All comments have been addressed

**********

publication criteria?

Reviewer #1: Yes

Reviewer #2: Yes

**********

3. Has the statistical analysis been performed appropriately and rigorously?

Reviewer #1: Yes

Reviewer #2: Yes

**********

4. Have the authors made all data underlying the findings in their manuscript fully available (please refer to the Data Availability Statement at the start of the manuscript PDF file)?

The PLOS Data policy

Reviewer #1: Yes

Reviewer #2: Yes

**********

5. Is the manuscript presented in an intelligible fashion and written in standard English?

Reviewer #1: Yes

Reviewer #2: No

**********

Reviewer #1: The authors have addressed concerns raised during previous review rounds. The manuscript demonstrates improvement in conceptual precision, methodological transparency, reporting quality, and interpretation of findings.

Reviewer #2: Thank you for addressing the previous review comments. The manuscript has improved substantially and now provides a clear and valuable contribution regarding physiotherapists’ preparedness to respond to gender-based violence across Spanish-speaking countries. The rationale, methodology, interpretation of findings, and discussion of limitations are generally appropriate and well presented.

I have only a few minor suggestions:

Please perform a final proofreading of the manuscript to correct a small number of typographical and formatting errors. For example:

"Sefl-reported preparedness" should be corrected to "Self-reported preparedness" (Results section) 402 line page-18.

",ore than one-third of respondents" should be corrected to "More than one-third of respondents" (Lived Experience of Gender-Based Violence section)363 line page-17.

Minor spacing and punctuation inconsistencies should be checked throughout the manuscript.

Although the handling of missing data has been explained, a brief statement summarizing participant flow and missing responses across major PREMIS domains would further improve transparency.

These are minor issues and do not detract from the overall quality of the work. The manuscript is scientifically sound, addresses an important public health issue and is suitable for publication after minor revision.

**********

what does this mean?). If published, this will include your full peer review and any attached files.

**Do you want your identity to be public for this peer review?**  If you choose “no”, your identity will remain anonymous but your review may still be made public.

For information about this choice, including consent withdrawal, please see our Privacy Policy

Reviewer #1: **Yes:**  Desmond Mathye

Reviewer #2: **Yes:**  Smati Sambyal

**********

[NOTE: If reviewer comments were submitted as an attachment file, they will be attached to this email and accessible via the submission site. Please log into your account, locate the manuscript record, and check for the action link "View Attachments". If this link does not appear, there are no attachment files.]

# Author comment: Author response to Decision Letter 3

**DOI:** 10.1371/journal.pgph.0007075.r006

22 Jun 2026

Attachment Submitted filename: Response_to_reviewers_auresp_3.docx

# Peer review: Decision Letter 3

**DOI:** 10.1371/journal.pgph.0007075.r007

**Authors:** Julia Robinson

22 Jul 2026

Physiotherapists are underutilised in health-sector responses to gender-based violence: a cross-sectional survey of preparedness across Spanish-speaking countries

PGPH-D-26-00627R3

Dear Prof Rosenbaum,

We are pleased to inform you that your manuscript 'Physiotherapists are underutilised in health-sector responses to gender-based violence: a cross-sectional survey of preparedness across Spanish-speaking countries' has been provisionally accepted for publication in PLOS Global Public Health.

Before your manuscript can be formally accepted you will need to complete some formatting changes, which you will receive in a follow up email. A member of our team will be in touch with a set of requests.

Please note that your manuscript will not be scheduled for publication until you have made the required changes, so a swift response is appreciated.

IMPORTANT: The editorial review process is now complete. PLOS will only permit corrections to spelling, formatting or significant scientific errors from this point onwards. Requests for major changes, or any which affect the scientific understanding of your work, will cause delays to the publication date of your manuscript.

If your institution or institutions have a press office, please notify them about your upcoming paper to help maximize its impact. If they'll be preparing press materials, please inform our press team as soon as possible -- no later than 48 hours after receiving the formal acceptance. Your manuscript will remain under strict press embargo until 2 pm Eastern Time on the date of publication. For more information, please contact globalpubhealth@plos.org.

Thank you again for supporting Open Access publishing; we are looking forward to publishing your work in PLOS Global Public Health.

Best regards,

Julia Robinson

Executive Editor

PLOS Global Public Health

***********************************************************

Reviewer Comments (if any, and for reference):

Reviewer's Responses to Questions

**Comments to the Author**

Reviewer #2: All comments have been addressed

**********

publication criteria?

Reviewer #2: Yes

**********

3. Has the statistical analysis been performed appropriately and rigorously?

Reviewer #2: Yes

**********

4. Have the authors made all data underlying the findings in their manuscript fully available (please refer to the Data Availability Statement at the start of the manuscript PDF file)?

The PLOS Data policy

Reviewer #2: Yes

**********

5. Is the manuscript presented in an intelligible fashion and written in standard English?

Reviewer #2: Yes

**********

**Reviewer #2:**  Thank you for addressing the previous review comments. The manuscript has improved considerably, and the requested corrections regarding typographical errors and clarification of missing data handling have been adequately addressed.

Overall, the manuscript is scientifically sound, addresses an important public health issue and is suitable for publication.

**********

what does this mean?). If published, this will include your full peer review and any attached files.

**Do you want your identity to be public for this peer review?**  If you choose “no”, your identity will remain anonymous but your review may still be made public.

For information about this choice, including consent withdrawal, please see our Privacy Policy

Reviewer #2: **Yes:**  Smati Sambyal

**********
